# Spatio-temporal modelling of *Culicoides* Latreille (Diptera: Ceratopogonidae) populations on Reunion Island (Indian Ocean)

**DOI:** 10.1186/s13071-021-04780-9

**Published:** 2021-05-27

**Authors:** Yannick Grimaud, Annelise Tran, Samuel Benkimoun, Floriane Boucher, Olivier Esnault, Catherine Cêtre-Sossah, Eric Cardinale, Claire Garros, Hélène Guis

**Affiliations:** 1 GDS Réunion, 1 rue du Père Hauck, 97418 La Plaine des Cafres, La Réunion, France; 2 University of Reunion Island, 15 avenue René Cassin, Sainte-Clotilde, 97715 La Réunion, France; 3 CIRAD, UMR ASTRE, Sainte-Clotilde, 97490 La Réunion, France; 4 ASTRE, University of Montpellier, CIRAD, INRAE, Montpellier, France; 5 CIRAD, UMR TETIS, Sainte-Clotilde, 97490 La Réunion, France; 6 TETIS, University of Montpellier, AgroParisTech, CIRAD, CNRS, INRAE, Montpellier, France; 7 CIRAD, UMR ASTRE, 101 Antananarivo, Madagascar; 8 Institut Pasteur of Madagascar, Epidemiology and Clinical Research Unit, Antananarivo, Madagascar; 9 FOFIFA DRZVP, Antananarivo, Madagascar

**Keywords:** *Culicoides*, Spatio-temporal distribution, Ocelet Modeling Platform, Reunion Island, Indian Ocean, Bluetongue, Epizootic hemorrhagic disease

## Abstract

**Background:**

Reunion Island regularly faces outbreaks of bluetongue and epizootic hemorrhagic diseases, two insect-borne orbiviral diseases of ruminants. Hematophagous midges of the genus *Culicoides* (Diptera: Ceratopogonidae) are the vectors of bluetongue (BTV) and epizootic hemorrhagic disease (EHDV) viruses. In a previous study, statistical models based on environmental and meteorological data were developed for the five *Culicoides* species present in the island to provide a better understanding of their ecology and predict their presence and abundance. The purpose of this study was to couple these statistical models with a Geographic Information System (GIS) to produce dynamic maps of the distribution of *Culicoides* throughout the island.

**Methods:**

Based on meteorological data from ground weather stations and satellite-derived environmental data, the abundance of each of the five *Culicoides* species was estimated for the 2214 husbandry locations on the island for the period ranging from February 2016 to June 2018. A large-scale *Culicoides* sampling campaign including 100 farms was carried out in March 2018 to validate the model.

**Results:**

According to the model predictions, no husbandry location was free of *Culicoides* throughout the study period. The five *Culicoides* species were present on average in 57.0% of the husbandry locations for *C.* *bolitinos* Meiswinkel, 40.7% for *C.* *enderleini* Cornet & Brunhes, 26.5% for *C.* *grahamii* Austen, 87.1% for *C.* *imicola* Kieffer and 91.8% for *C.* *kibatiensis* Goetghebuer. The models also showed high seasonal variations in their distribution. During the validation process, predictions were acceptable for *C.* *bolitinos*, *C.* *enderleini* and *C.* *kibatiensis*, with normalized root mean square errors (NRMSE) of 15.4%, 13.6% and 16.5%, respectively. The NRMSE was 27.4% for *C.* *grahamii*. For *C.* *imicola*, the NRMSE was acceptable (11.9%) considering all husbandry locations except in two specific areas, the Cirque de Salazie—an inner mountainous part of the island—and the sea edge, where the model overestimated its abundance.

**Conclusions:**

Our model provides, for the first time to our knowledge, an operational tool to better understand and predict the distribution of *Culicoides* in Reunion Island. As it predicts a wide spatial distribution of the five *Culicoides* species throughout the year and taking into consideration their vector competence, our results suggest that BTV and EHDV can circulate continuously on the island. As further actions, our model could be coupled with an epidemiological model of BTV and EHDV transmission to improve risk assessment of *Culicoides*-borne diseases on the island.

**Graphic Abstract:**

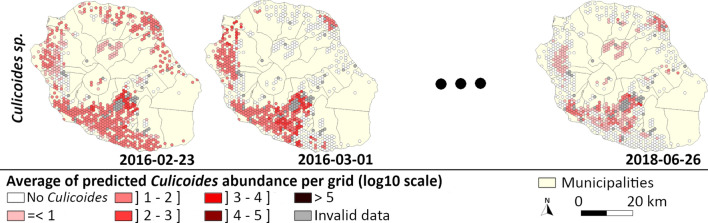

**Supplementary Information:**

The online version contains supplementary material available at 10.1186/s13071-021-04780-9.

## Background

Temporal and spatial variations of the climate and environment have an impact on vector populations and on the transmission of vector-borne diseases (VBDs) [1–3]. For vector-borne transmission of an infectious agent to occur, host(s), vector(s) and the infectious agent must interact in an enabling environment [1]. Sufficient interaction is based on quantitative characteristics related to the populations of the three actors (host densities, abundance, aggressivity, longevity and infectivity of the vectors, number of infectious agents in the host) and the behaviors that favor frequent contact between these populations [1, 4, 5]. The environment and meteorological parameters play a decisive role in the vector system. For transmission to occur they need to (i) allow replication of the infectious agent within the vector, (ii) provide an adequate biotope for hosts and vectors and (iii) provide conditions that allow contact between hosts and vectors [6]. They can act on different spatial and temporal scales [2] and with different levels of impact [7]. Also, because the environment and climate are heterogeneous in time and space, the joint presence of the three actors of the vector triad, and ultimately the transmission of the infectious agent, is only possible over specific spaces and periods. Quantifying vector populations and their variations is therefore a fundamental approach to understanding VBDs. Statistical models help understand the interactions between climate-environment and vectors-VBDs [8]. When these models are incorporated into Geographic Information Systems (GIS), spatially explicit outputs such as distribution maps can be produced, making it possible to establish and rank the risk of exposure to vector bites and associated pathogens in a given geographical area [9, 10]. This approach is particularly relevant for areas where surveillance data are lacking and the level of risk is unknown, as it provides a useful tool for health policy makers and vector-control agencies and the public [9].

Some biting midge species of the genus *Culicoides* are vector species of economically important viruses affecting livestock [11]. Because these viruses are mainly transmitted to hosts by bites of *Culicoides* [12], their distribution and the intensity of infection are dependent on the distribution and abundance of their vectors [13, 14]. Epizootic events of bluetongue (BT), epizootic hemorrhagic disease (EHD) and African horse sickness (AHS), three *Culicoides*-borne viral diseases, respectively, in Europe [15–17], the Mediterranean basin [18, 19] and Africa [20, 21] have highlighted the need to map vector distribution and produce detailed predictive risk maps of *Culicoides*-borne diseases. Consequently, during the last decades, various studies conducted in Europe and Africa have mapped occurrence or abundance of *Culicoides* at a continental or country-wide scale [22–37] and less commonly at a local scale [8, 38, 39]. Variations in the demographics and blood-feeding behavior of arthropod vectors, resulting from the temporal distribution of species-specific environmental thresholds, can impact pathogen transmission and ultimately disease emergence [40]. Hence, a spatio-temporal approach is particularly useful when the risk of exposure to vectors or VBDs is clustered in time and space [9]. This approach was favored by different authors [41–44].

Five *Culicoides* species have been recorded in Reunion Island [45, 46]: *C.* *bolitinos*, *C.* *enderleini, C. grahamii*, *C.* *imicola* and *C.* *kibatiensis*. Studies on trophic preferences or on the origin of blood meals showed that all these species are associated with cattle, small ruminants and/or horses [47–50]. In addition, *C. bolitinos*, *C. imicola* and *C. enderleini* have been found naturally infected by BTV in Africa [51–53], and the vector competence of the first two towards BTV and EHDV has been demonstrated [54, 55]. These elements show that each of the five species fulfills at least one of the three criteria (host contact, natural infection and vector competence) characterizing a vector according to the WHO [56] and that all of them may therefore play a role in the circulation of *Culicoides*-borne virus in Reunion's livestock. Since the first detection of bluetongue virus (BTV) in 1979 [57] and epizootic hemorrhagic disease virus (EHDV) in 2003 [58] in Reunion Island, enzootic circulation of the former and epizootic circulation of the latter have been recorded [59]. In parallel, a serological survey conducted in 2011 [59] showed that: (1) two thirds of the cattle tested were EHDV positive and distributed throughout the island; (2) nearly 80% of cattle, 50% of goats and 21.5% of sheep were seropositive, suggesting a high level of BTV circulation among these animals. Furthermore, clinical cases were almost exclusively reported during or after the rainy season (November–April), and in the most recent cases (years 2016, 2018 and 2019) they were reported mainly on farms at high altitudes (GDS Réunion personal communication). The seasonality of these clinical cases coincided with the high abundance periods of *C. imicola*, *C. bolitinos* and *C. enderleini* observed in [46]. However, the location of these clinical cases was more consistent with the distribution of mid- and high-altitude species such as *C. kibatiensis*, *C. grahamii* and *C. bolitinos* [45]. In any case, the distribution throughout the island of EHDV or BTV positive animals suggests the involvement of low- and high-altitude species but the level of involvement of each of the five species in the circulation of the two orbiviruses remains unknown. The potential vector role of each species and their spatial and/or temporal congruence with the two viruses highlight the necessity to develop dynamic distribution maps of *Culicoides* in Reunion Island.

Statistical models were recently developed for each *Culicoides* species on Reunion Island [46] to provide a better understanding of their ecology and determine their periods of high abundance based on meteorological and environmental data. This first study focused on the temporal dimension of the *Culicoides* dynamics, but the spatial dimension was not addressed. The aim of this study is to couple previously developed statistical temporal models of *Culicoides* dynamics [46] with GIS techniques to provide spatio-temporal vector abundance maps for each *Culicoides* species in Reunion Island. It aims at addressing the needs expressed by different stakeholders, including breeders and veterinary services, to identify periods and areas when and where prevention and vector control strategies could be targeted.

## Methods

### Study sites

**Fig. 1:**
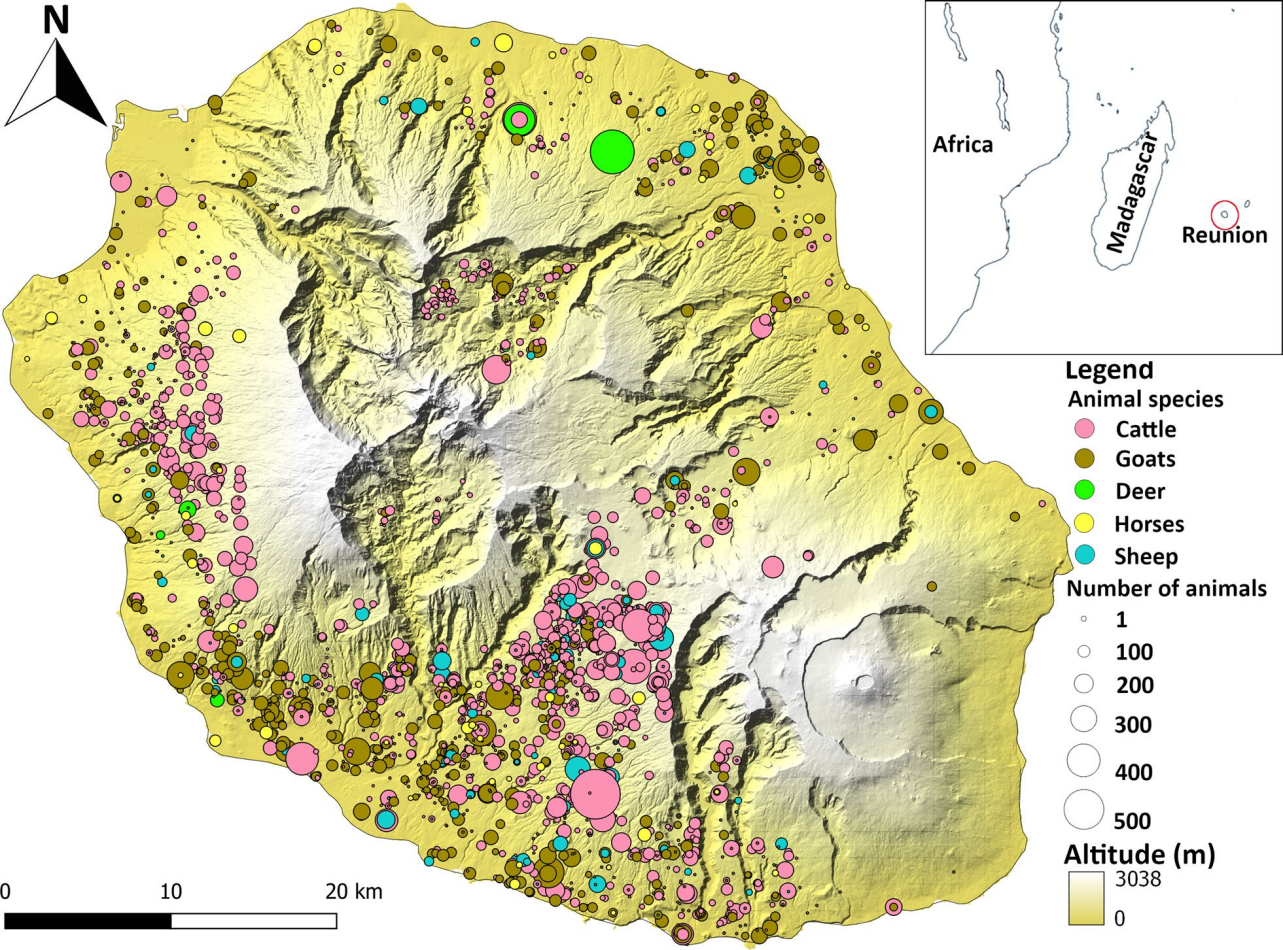
Spatial distribution of cattle, goats, sheep, deer and horses on Reunion Island

The study area is Reunion Island, a French department located in the southwestern Indian Ocean. This mountainous island rises to 3069 m and has a tropical climate with high annual rainfall (2000–8000 mm) on the windward (east) coast and drier weather (600–2000 mm) on the leeward (west) coast [60]. The temperature is linked to altitude and ranges from an average of 26 °C on the coasts to less than 12 °C at over 2000 m [60]. A warm rainy season (austral summer) from December to mid-April and a cooler dry season (austral winter) between mid-April and November are observed. The highest concentrations of dairy cattle and suckler farms are found in the western and southwestern highlands, where temperatures are more temperate. The study sites include all the livestock (cattle, small ruminants, deer and horse) farms of the island. Livestock units are defined by the association of one breeder, one type of animal and one type of production (dairy farms, fatteners or pasture farms). In accordance to the 2016–2017 national census database and Groupement de Défense Sanitaire de La Réunion (GDS Réunion survey, the main association of breeders of the island) survey, the island comprises 2560 livestock units (Fig. 1) of cattle (1337), goats (980), sheep (174), deer (12) and horses (57) distributed among 2070 breeders. For the model, livestock units occupying the same production space, whatever the type of animal, were considered as a single geographic unit. Conversely, geographically separated livestock units of the same breeder were considered as separate units. Therefore, 2214 husbandry locations with distinct geographical coordinates, composed of single or multiple livestock units, were defined and used as elementary geographic units in the *Culicoides* abundance model.

### Meteorological and environmental data

The temporal dynamics models developed for each *Culicoides* species [46] include 11 categories of meteorological and environmental variables to estimate abundance (Table 1).

**Table 1:** Data sources for the 11 categories of variables. Time-dependent variables (i.e. temperature, humidity, rainfall, wind speed, global radiation and vegetation index) were obtained for the January 2016 to June 2018 period

Variable categories	Measurement location	Data information	Sources
*Time-dependant variables*			
Temperature (31)*	From the nearest meteorological station	Correction of the temperature at the husbandry location to take into account the difference in altitude with the meteorological station	Location of meteorological station: https://publitheque.meteo.fr Daily data: www.smatis.re Altitude: BD ALTI®: http://professionnels.ign.fr/bdalti Roughness categories of the land around the station: https://sites.google.com/site/venturiec1/calendar
Humidity (18)*		29.2% of missing data	
Rainfall (31)*			
Wind speed at 2 m above the ground (30)*		24.1% of missing data; correction of the wind data provided at 10 m above the ground according to the roughness of the landscape according to [46]	
Global radiation (28)*			
Vegetation index (NDVI)	At site location	MODIS Terra 16-day composite images; 250 m spatial resolution	Product MOD13Q1 [61]; https://lpdaac.usgs.gov/
*Non-dynamic variables*			
Eco-climate area	At site location		Map of “Urban Planning and Native Plants Approach” (DAUPI): http://daupi.cbnm.org/palette/#/taxons)
Land use	Buffer area		2016–2017 land-use map [62, 63]: http://aware.cirad.fr
Husbandry location density			2016–2017 national and GDS Réunion census databases
Animal density			
Building opening size	At site location		

^*^Number of meteorological stations recording the data

Missing data for wind (24.1%) and humidity (29.2%) were estimated by time linear interpolation using “na.approx” function in zoo package [64].

### Models of *Culicoides* presence and abundance

For each husbandry location, the abundance of each *Culicoides* species from February 2016 to June 2018 was estimated using the temporal dynamics models developed in [46]. This estimated abundance reflects the number of *Culicoides* that could have been captured near the animals by an OVI (Onderstepoort Veterinary Institute) light trap overnight. The temporal models are hurdle models, i.e. a presence/absence model (logit-link logistic regression model) combined with an abundance model (log-link mixed-effect zero-truncated negative binomial model), built from a 2-year dataset of biweekly catches in 11 farms.

All variables and coefficients used to estimate *Culicoides* abundances are presented in Additional file 1: Tables S1 to S5.

To apply the models to the scale of Reunion Island, the random effect of farms in the count part of the hurdle model was neglected.

The environmental characteristics of the 11 farms used to build the temporal model define their validity domain: no predictions can be made on the husbandry locations with values of environmental characteristics outside the range of values of the predictive variables. The limiting variables are presented in Additional file 1: Tables S1 to S5.

## Preprocessing

The eco-climate area corresponding to each husbandry location, the husbandry location density, animal density and the percentage of land use coverage in different buffer sizes (0.5 km, 1 km or 2 km) around each husbandry location were all extracted using QGIS [65]. The altitude was extracted for each husbandry location and each weather station using QGIS. Building opening size was interpreted according to the general configuration of the type of farming observed in Reunion Island: from 0 to 25% for dairy farms, from 25 to 100% for fatteners and enclosure for pasture farms.

### Spatio-temporal dynamics

The representation of the spatio-temporal dynamics of *Culicoides* was built with Ocelet language and open modeling platform (www.ocelet.org; Ocelet codes are freely available on the CIRAD Dataverse). Ocelet is an open-access domain-specific language and simulation tool for modeling changes in geographical landscapes and facilitating the processing of geographical information [66].

**Fig. 2:**
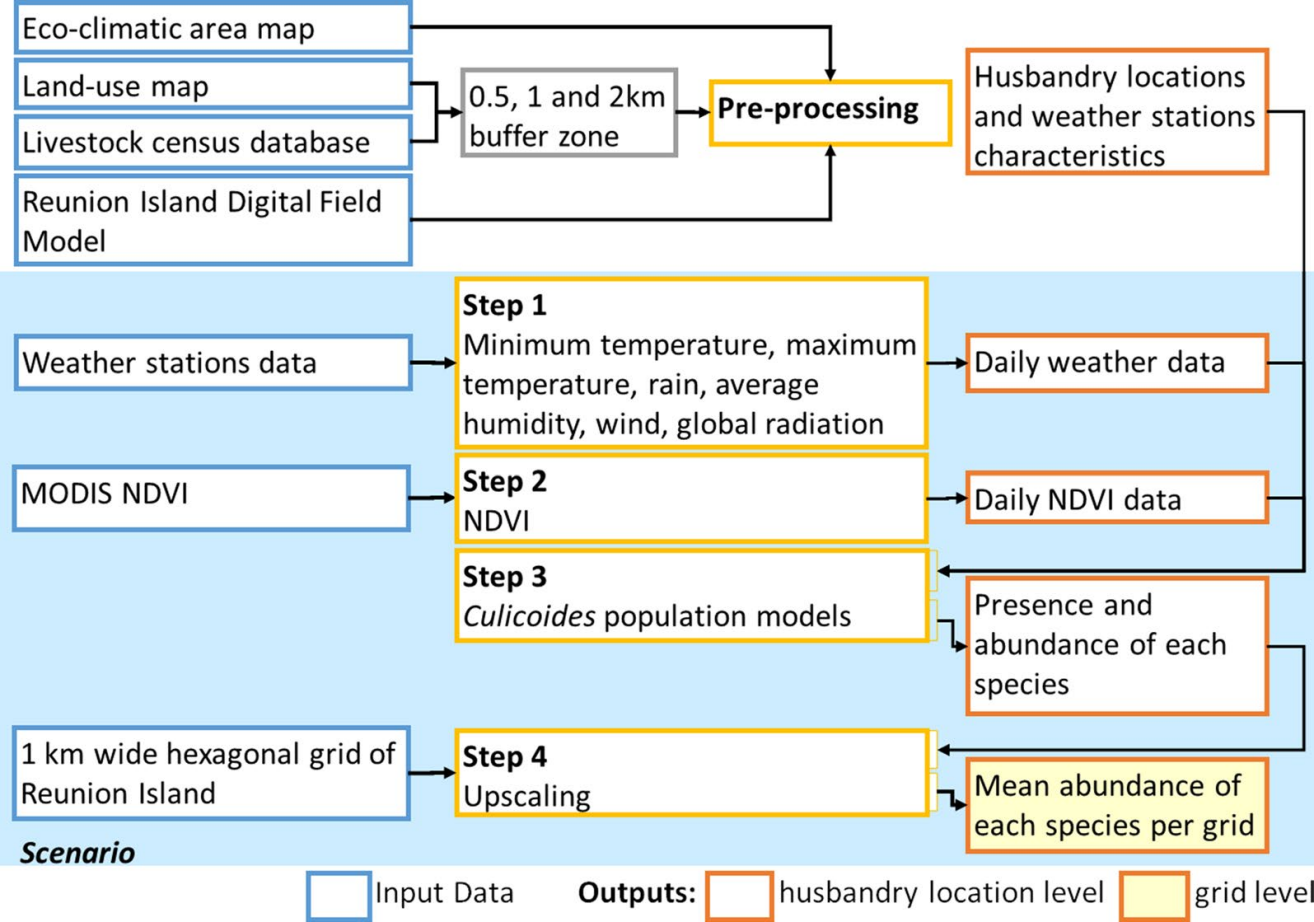
Workflow diagram for the application at Reunion Island scale of the *Culicoides* spatio-temporal models

Our model comprises four main elements called “entities”: (i) the 2214 husbandry locations (point geometry), characterized by their respective values of eco-climatic area, land use, husbandry location density, animal density and building opening size, (ii) the weather stations (point geometry) whose daily minimum and maximum temperatures, rainfall, wind speed, global radiation and humidity are imported as text files (csv format), (iii) the satellite-derived vegetation indices, imported as raster data, and (iv) a 1-km-wide hexagonal grid to map model output (Fig. 2). The entities interact through spatial relations: for each husbandry location, the values of the meteorological variables were defined as those of the closest weather station, and the NDVI value was defined as the NDVI of the pixel in which the husbandry location was contained.

The scenario, which defines the sequence of operations and interactions between entities was defined as follows (Fig. 2). Step 1: daily meteorological variables were read and attributed to each corresponding husbandry location. A correction of − 0.0075 °C m^−1^ [67] was applied to the temperature based on the altitude difference in meters between the station and the husbandry location. A correction of the 10 m above the ground wind speed measures was applied to convert them into 2 m above the ground wind speed estimates [46]. Step 2: daily NDVI values for each husbandry location were either read from a raster file if the date corresponded to the date of acquisition of MODIS NDVI or estimated using a temporal linear interpolation [68]. When the pixel corresponding to a husbandry location had a NDVI value lower than zero (suggesting that the pixel was masked by a cloud), the positive value of the nearest pixel was selected. Step 3: for each husbandry location, the probability of presence and the predicted abundance of each species were computed provided they were within the validity limits of the model variables. Step 4: for each hexagonal grid, the abundance of *Culicoides* species was computed as the average values of *Culicoides* abundance of the husbandry locations it contained.

### Validation

To validate the simulation of the spatial dynamics of each species, an entomological survey was carried out at the scale of the whole island. A total of 100 farms distributed throughout the island were sampled representing 101 catches (one farm was sampled at two husbandry locations). The 11 farms used to build the temporal model in [46] were included in the survey. Single night catch collection was conducted from 7 to 22 March 2018 using OVI traps. The trapping and identification of *Culicoides* species were the same as described in [46].

Observed and predicted values at the date of capture were compared using the area under the curve (AUC) of the ROC (receiver-operating characteristic) curve [69] for presence/absence and the normalized root mean square error (NRMSE) standardized to the range of observations for abundance. These calculations were performed using R version 3.4.1 [70] with pROC [71] and hydroGOF [72] packages. Standardized residuals, i.e. the difference between a prediction and an observation divided by the range of observed values, were also mapped to identify clusters of correct predictions, overestimations or underestimations.

## Results

**Fig. 3:**
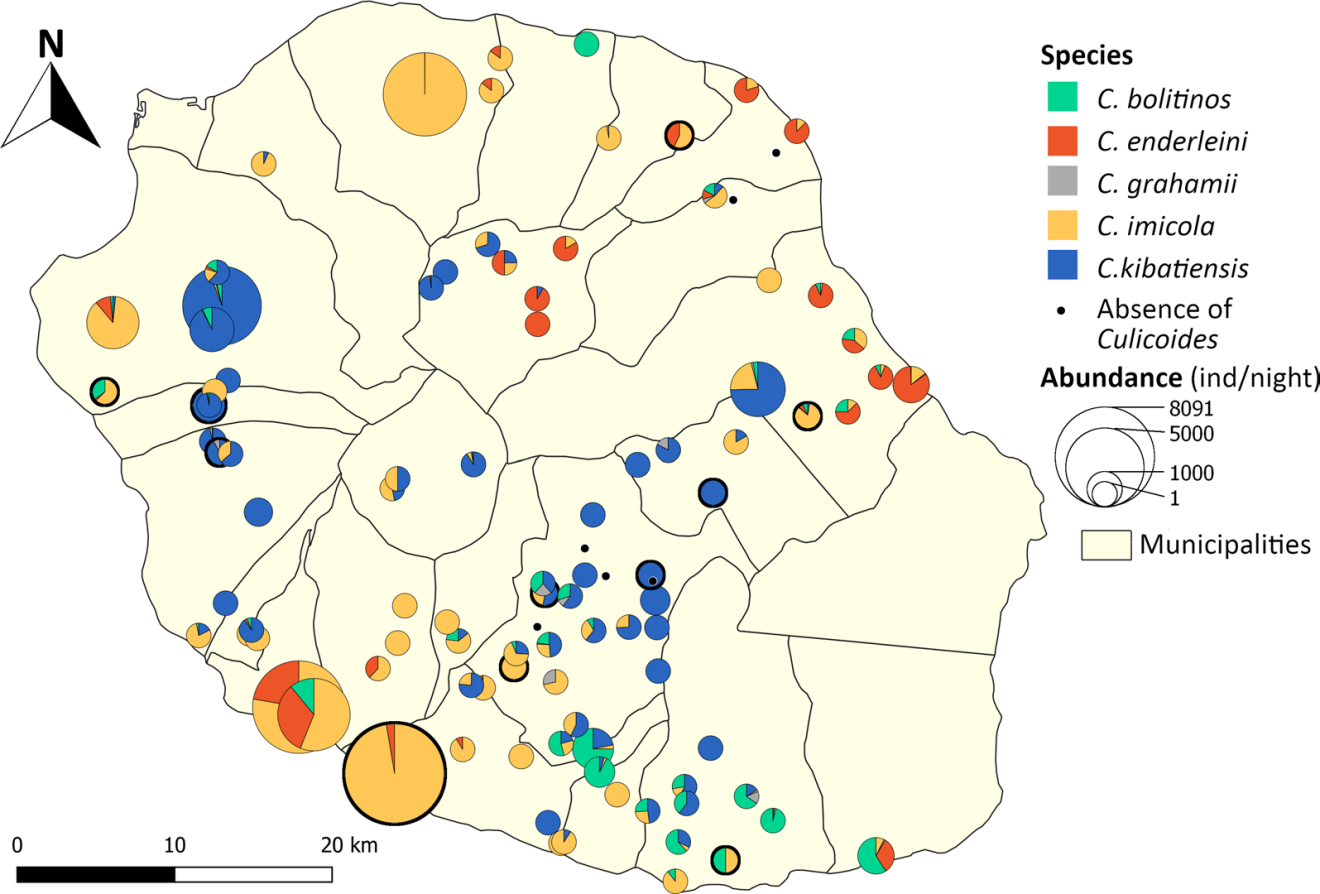
Spatial distribution of *Culicoides* species observed during the March 2018 sampling campaign. The bold circles correspond to the 11 farms used to build the temporal model in [46]

The survey conducted in March 2018 (Fig. 3) confirmed the presence of the five species identified by [45]. A total of 50,526 *Culicoides* were caught during the 101 nights of the trapping campaign with a maximum of 8091 individuals in single night of trapping. No *Culicoides* were caught in six farms. For each species, the total number of individuals, mean and number of positive catches in brackets was: 4087 (40.5; 41) for *C.* *bolitinos*, 5314 (52.6; 29) for *C.* *enderleini*, 154 (1.5; 20) for *C.* *grahamii*, 26,908 (266.4; 69) for *C.* *imicola* and 14,063 (139.2; 61) for *C.* *kibatiensis* (Additional file 2: Table S6). Spatial heterogeneity was observed in the abundance of species. *Culicoides imicola* and *C. enderleini* were found mainly at low altitudes. However, *C. imicola* was clearly more abundant than any other species on the north, west and southwest coasts and was also widely distributed as it was also found in the higher parts of the island. On the east coast, *C. enderleini* appeared as the dominant species at six of the seven lowest sites in altitude despite low abundance. *Culicoides bolitinos* was abundant on a strip from the south coast to the southwestern heights of the island. Finally, *C. grahamii*, and *C. kibatiensis* were found mainly at high altitudes where the latter was dominant and widespread.

The total number of husbandry locations on which it was possible to estimate abundances (i.e. their environmental characteristics were within the validity domain of the model) was 1807 (81.6%) for *C.* *bolitinos*, 1534 (69.3%) for *C.* *enderleini*, 2178 (98.4%) for *C.* *grahamii*, 1866 (84.3%) for *C.* *imicola* and 1922 (86.8%) for *C.* *kibatiensis*, out of the 2214 husbandry locations in the sample size.

During the 28 months of spatio-temporal dynamics simulation, *C.* *kibatiensis* and *C.* *imicola* were the most widespread species, present on average (minimum–maximum) in 91.8% (82.0–99.8%) and 87.1% (63.7–99.1%) of the husbandry locations, respectively. In contrast, *C.* *grahamii* and *C.* *enderleini* were the least widespread species, present on average in 26.5% (6.8–74.0%) and 40.7%of (17.3–64.3%) husbandry locations, respectively. *C.* *bolitinos* was present on average in 57.0% (14.0–87.4%) of the husbandry locations.

Estimated abundances remained generally low. Considering times points when the species were present, the medians of estimated abundances (*Culicoides* per trap per night), first and third quartiles in brackets, were 12.1 (5.7–23.7) for *C.* *bolitinos*, 0.2 (0.03–1.1) for *C.* *enderleini*, 1.7 (0.5–3.7) for *C.* *grahamii*, 23 (1.2–148) for *C.* *imicola* and 3.7 (1.2–12.1) for *C.* *kibatiensis*. However, *C. imicola* was estimated to have a high abundance in an important number of husbandry locations compared to other species. On average for *C.* *imicola*, more than 1000 individuals were estimated on 13.7% of the husbandry locations. This rate dropped below 0.1% for the other species. Considering a lower expectation of 100 individuals, the rate was 28.3% for *C.* *imicola* and less than 2.4% for the other species.

**Fig. 4:**
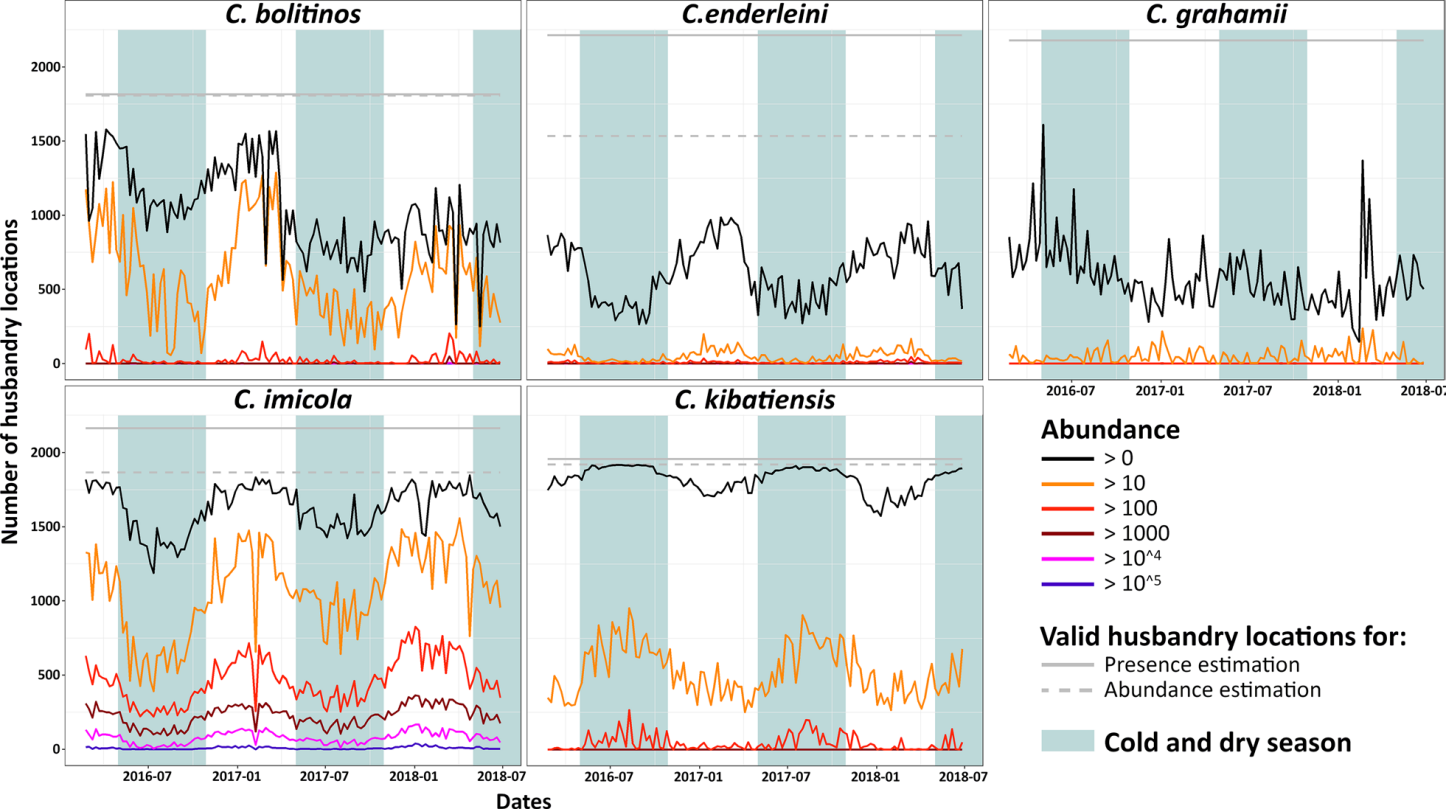
Temporal variations in the number of husbandry locations with an estimated positive abundance for each of the *Culicoides* species. Husbandry locations with more than 10^^1^ to 10^^5^ individuals are also shown

A clear seasonal pattern was observed for all species (Fig. 4). *Culicoides bolitinos*, *C.* *enderleini* and *C.* *imicola* were present in fewer husbandry locations during the cold and dry season than during the hot and rainy season. These seasonal patterns could also be observed when considering husbandry locations with > 10 *Culicoides*. The opposite situation with an increased presence in study sites in the cold dry season was observed for *C.* *kibatiensis* and, to a lesser extent because seasonal patterns were harder to identify, for *C.* *grahamii*.

Interestingly, *C. bolitinos* was globally present in more sites during the period from early 2016 to the end of the 2016–2017 hot and rainy season than during the period from the end of the 2016–2017 hot and rainy season to mid-2018. In the former period, the average percentage (range in brackets) of positive husbandry locations was 69.5% (37.2–87.4%), and during the latter, it decreased to 45.8% (14.0–66.7%). A difference was also noticeable between the two cold seasons of 2016 and 2017 for *C. imicola* when considering sites with more than ten individuals and for *C. grahamii*. On average, more than ten individuals of *C. imicola* were predicted on 35.8% (20.4–58.9%) of husbandry locations during the cold season of 2016 compared to 50.4% (33.4–60.1%) during the cold season of 2017. For *C. grahamii*, the average percentages of positive husbandry locations were 38.0% (24.9–86.3%) and 28.9% (16.0–41.1%) for the same two seasons.

**Fig. 5:**
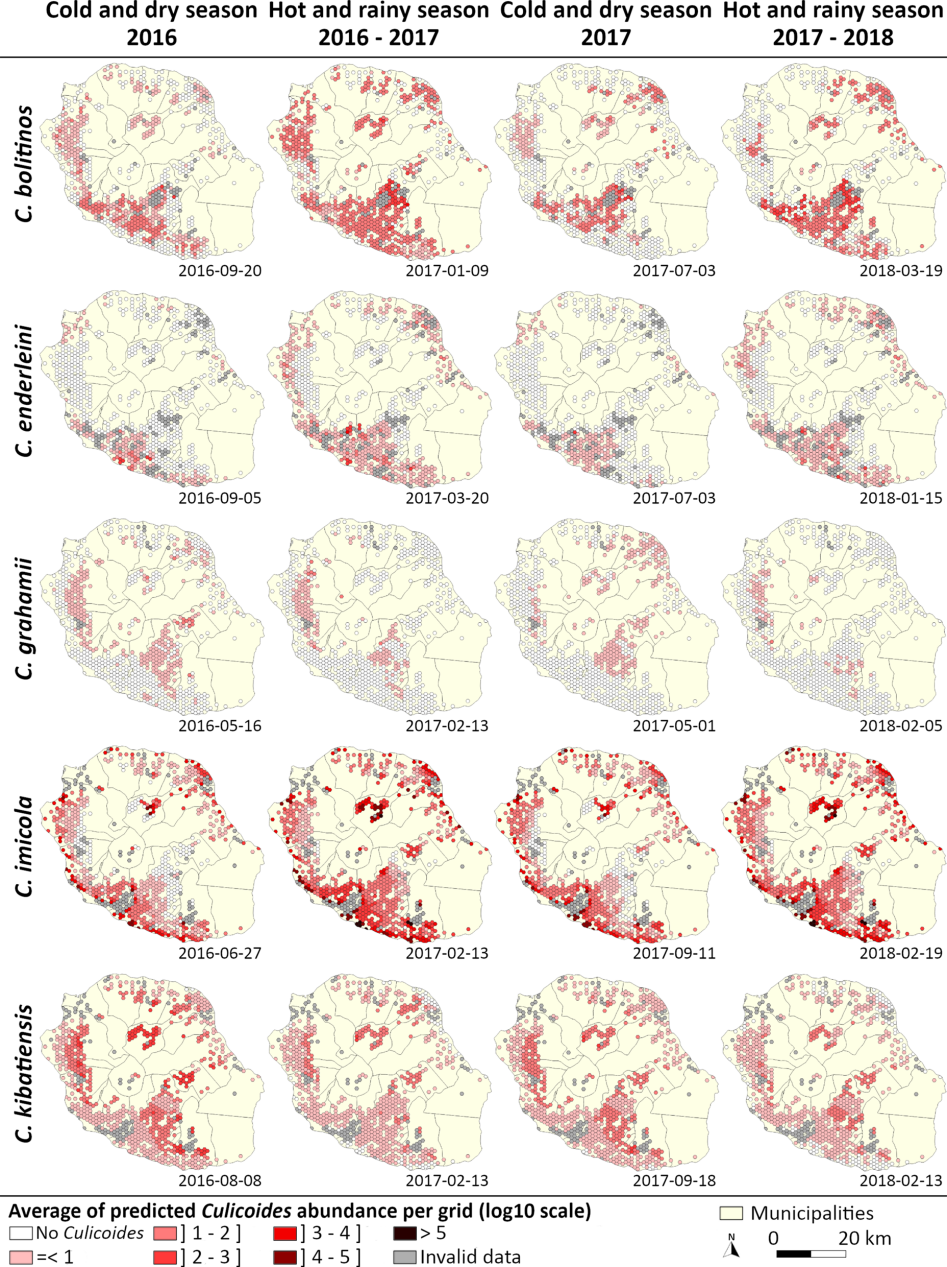
Example of modeled density maps of each *Culicoides* species, Reunion Island, 2016–2018. The examples chosen are those closest to the third quartile of the number of positive husbandry locations during the hot and rainy season for *C.* *bolitinos*, *C.* *enderleini* and *C.* *imicola* and during the cold season for *C.* *grahamii* and *C.* *kibatiensis*. For the opposite seasons, the examples are the closest to the first quartile

The abundance maps produced with a 7-day time step showed the spatial and temporal variation for each species (see examples of abundance maps in Fig. 5; Additional files 3, 4, 5, 6 and 7 for movies). According to the model, *C.* *imicola* occupied the island’s coastal areas continuously and with high abundance. During the hot and rainy season, its distribution expanded and reached sites located further from the coast toward the interior of the island.

Climatic and environmental conditions were favorable for the distribution of *C.* *kibatiensis* throughout the island, except on a thin southern and northeastern coastline during the hot and rainy season. However, areas of high abundance were only found in the highlands.

*Culicoides bolitinos* occupied a wide area from the coastal area to the interior of the island although the southern and western coastal areas become less favorable to its presence during the cold and dry season. The highest abundances were estimated for the mid- and high-altitude husbandry locations. The results also highlighted a high inter-annual variability, with a distribution of *C.* *bolitinos* much more restricted and less abundant in the western area in 2017 than in 2016.

*Culicoides enderleini* occupied the coastal areas of the island, and during the cold and dry season, it persisted only in the southwest and in a small area to the northwest. Conversely, *C.* *grahamii* very clearly occupied the western and southwestern highlands, and its distribution could extend to the northeast during the cold and dry season.

**Fig. 6:**
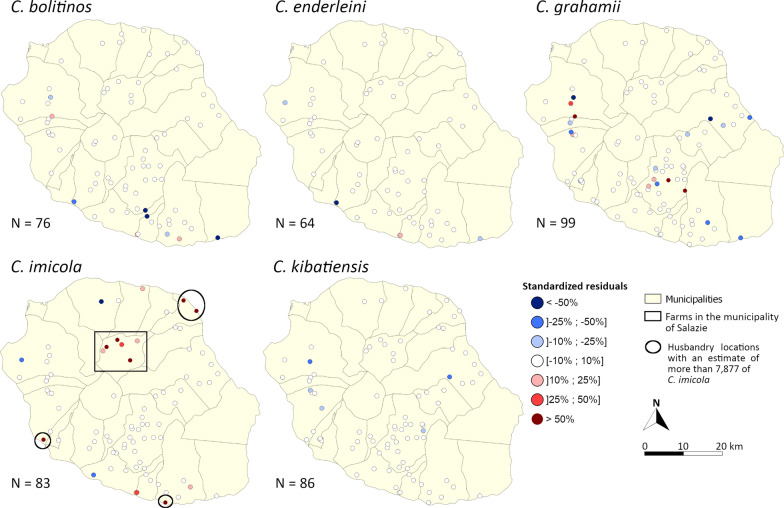
Maps of standardized residuals between predictions and observations from the March 2018 sampling campaign

Predictive accuracy of presence and absence was acceptable for *C.* *imicola* and *C.* *kibatiensis* with ROC AUCs of 0.755 and 0.730, respectively. For *C.* *bolitinos*, *C.* *enderleini* and *C.* *grahamii*, predictions of presence were not as good with AUCs of 0.557, 0.649 and 0.588, respectively. Regarding abundance estimates, the NRMSEs were 15.4% for *C.* *bolitinos*, 13.6% for *C.* *enderleini*, 27.4% for *C.* *grahamii* and 16.5% for *C.* *kibatiensis*. Considering all husbandry locations, the abundance predictions for *C.* *imicola* showed a high NRMSE of 252.5%. This high NRMSE value can be explained by a very large difference in values, exacerbated by an exponential function in the models, between observations and predictions at a few sites. Indeed, the maps of standardized residuals (Fig. 6) showed a high variability between predictions and observations for *C.* *imicola* at the Salazie (inner mountainous area) husbandry locations and at four other husbandry locations located in the south, southwest and northeast seacoasts, with a tendency to overestimate. Without considering the Salazie husbandry locations and the four other ones for which the model predicted > 7877 individuals per trap per night (maximum observation for *C.* *imicola*), the NRMSE was 11.9%.

The highest variabilities between observations and predictions were observed in the south and west for *C.* *bolitinos* and on the south coast for *C.* *enderleini*. For *C.* *grahamii*, a strong variability seemed to exist between the observations and the predictions. Finally, predictions for *C.* *kibatiensis* lead to underestimation of abundance, mainly in the western highlands.

## Discussion

BTV and EHDV epidemics can cause severe economic losses to farmers [12, 18]. In Reunion Island, both a large-scale inventory and longitudinal monitoring allowed the identification of local *Culicoides* species, improving the understanding of their ecology and modeling high abundance periods [45, 46]. The present study provides, for the first time to our knowledge, a spatial model of the abundance of *Culicoides* in Reunion Island, designed to implement an operational tool to help stakeholders and farmers to identify and communicate on disease risk periods and areas.

Overall, the predicted spatio-temporal distributions of each *Culicoides* species coincide with the main observations made by [45] and [46]: (i) low overall abundance, except for *C.* *imicola* in coastal areas; (ii) higher abundances of *C.* *imicola* and *C.* *enderleini* at low altitude and during the hot and rainy season; (iii) higher abundance of *C.* *bolitinos* at mid-altitude and during the hot and rainy season; (iv) higher abundances of *C. kibatiensis* and *C.* *grahamii* at high altitude and during the cold and dry season. Our modeling results showed that *C.* *imicola* and *C.* *kibatiensis* have a wide spatial distribution and that there is virtually not a single husbandry location that would not be affected by *Culicoides*. This prediction was verified by data from the March 2018 campaign where *Culicoides* were found in 95 out of the 101 catches. This confirms that *Culicoides* species can occupy a wide variety of climates encountered on Reunion Island as already mentioned for the Palearctic region [11].

The spatial distribution of species observed during the March 2018 survey also confirmed the global distribution in the island described by Desvars et al. [45] and Grimaud et al. [46]. However, the greater sampling effort made and the use of the reference trap (OVI) enabled detecting a larger distribution of *C.* *bolitinos* in the south of the island and of *C.* *enderleini* on the northeast coast and on the highlands. A more extensive spatial distribution was also observed for *C. imicola* and *C. kibatiensis*, which is consistent with the predicted spatio-temporal distribution (Fig. 5).

Together the wide permanent distribution of *Culicoides* and their known vector competence [52, 55] suggest that BTV and EHDV can circulate throughout the year and throughout the island in vector populations. However, each species showed spatial and temporal variations in abundance suggesting different implications in the transmission of the two viruses. As Donnelly et al. [73] pointed out, locally high abundance, although seasonal, and wide distribution are conditions characterizing the primary role of a vector. Therefore, knowing these variations in abundance and distribution makes it possible to develop scenarios on how *Culicoides* species take turns to ensure a continuum of BTV and EHDV transmission across the island, but also to identify which species might be the most involved during an epizootic.

Given the close link between transmission and biting rates, it would be tempting to position *C.* *imicola* as the main vector of BTV and EHDV. Indeed, the four other species reach abundances of > 100 individuals in an average of 2.4% of husbandry locations and > 1000 individuals in only 0.1% of husbandry locations on average, suggesting that their participation in transmission may remain low compared to that of *C.* *imicola*. However, other parameters such as virus replication, host preference, biting rate and longevity should be more carefully assessed to determine the precise roles in virus transmission of the different species [54]. A concrete example is *C.* *bolitinos*, whose biology was closely associated with cattle and more adapted to cold environments than *C.* *imicola*, is a vector of prime importance during winter and in the coldest parts of South Africa [54, 74]. If, due to their abundance, *C.* *imicola* and *C.* *kibatiensis* are the ideal candidates in coastal areas and highlands, respectively, it is not unreasonable to think that *C.* *bolitinos* could be the first-rate vector at higher altitudes in Reunion Island. The importance of the two other species (*C.* *enderleini* and *C.* *grahamii*) cannot be ruled out on the sole assessment of their abundance without assessing their vector competence.

Interannual variations in the distribution and/or abundance of *C. bolitinos*, *C. imicola* and *C. grahamii* have also been highlighted thanks to 2 years of spatio-temporal dynamics simulation. Fluctuations in seasonal patterns of demographic parameters and phenology (duration of presence on husbandry location) of *Culicoides* can modify the risk of orbivirus transmission [40, 75], and coincidences can be noted in Reunion Island. Indeed, *C. bolitinos* showed strong interannual variability resulting in a higher abundance and distribution in the western highlands during the 2016 warm season than in 2017 and 2018. This period and area coincided with the clinical cases of EHDV in 2016 suggesting that the increase in *C. bolitinos* abundance in some years could amplify the transmission of EHDV. Interannual changes in the occurrence of clinical cases have also been pointed out by other authors [76–79]. In addition, annual and interannual variations in climatic conditions can also impact the specific composition, leading to a change in the circulation of pathogens. The co-occurrence of competent vectors could increase the circulation potential of a pathogen by deploying a range of different responses to environmental climatic factors and to the hosts present. Quaglia et al. [40] showed that *Culicoides* communities can vary beyond simple seasonal variations and that the dynamics of these changes play an important role in the epidemiology of EHD in Florida. Hence, the presence of several vector or potential vector species, their wide distributions, annual and interannual change in demographic parameters, and different trophic preferences and behaviors reinforce the need to better understand the BTV and EHDV risk on Reunion Island. The calculation of the basic reproduction rate R_0_ would be a very interesting approach to assess this risk [42, 80]. A community-based approach could also be a way to improve the understanding of EHDV and BTV transmission risk in Reunion Island.

The abundance of each *Culicoides* species was estimated from mixed-effect zero-truncated negative binomial models developed in a previous study [46]. To apply these models to the entire island, the random effect of farms was neglected, as mixed models do not allow predictions on subjects that were not part of the original training data. However, the standard deviation of the random effects for farms provided in Grimaud et al. [46] was negligible for all species except *C.* *kibatiensis*. Consequently, we can consider that the abundance estimates of *C.* *bolitinos*, *C.* *enderleini*, *C.* *grahamii* and *C.* *imicola* were not biased by the intrinsic effects of farms and were under the almost exclusive governance of climate and environment. However, for *C.* *kibatiensis*, the random effect of farms could not be considered as negligible. The modeled dynamics of *C.* *kibatiensis* allowed identifying the favourable areas according to climatic and environmental data only, but intrinsic characteristics of the farm could greatly impact these abundances.

When comparing modeled abundance predictions with field observations from the March 2018 survey, predictions in species presence were acceptable for the two most common species, *C.* *imicola* and *C.* *kibatiensis*. For the three other species (*C.* *bolitinos*, *C.* *enderleini* and *C.* *grahamii*), presence predictions were less fit. Regarding abundance estimates, predictions were acceptable for *C.* *bolitinos*, *C.* *enderleini* and *C.* *kibatiensis*, but less good for *C.* *grahamii* and *C.* *imicola*.

It should be noted that the high variability of catches is a general problem in vector modeling [41]. As Baylis et al. pointed out [26], the number of *Culicoides* that can be caught per night using a light trap depends on the size of the local *Culicoides* population, the activity rate and the efficiency of the trap; the latter two are themselves affected by the local weather conditions. According to the same study, the absence of repeated captures over a short period of time reduces the accuracy of presence estimation, especially when *Culicoides* abundance is low. Also, the catches were made after a mild hurricane event that occurred on 5 March 2018, which still resulted in heavy rainfall (http://www.meteofrance.fr/actualites/59989790-un-point-sur-le-cyclone-dumazile). Thus, the model predictions were compared to 1-night catch data of March 2018, potentially impacted by the previous hurricane event, which could explain the lack of accuracy in the prediction of presence for the three species with the lowest observed abundances (*C.* *bolitinos*, *C.* *enderleini* and *C.* *grahamii*). It should be noted that two mild hurricane events affected Reunion Island with heavy rains and moderate winds in February 2017 and January 2018 (https://reunion-extreme.re/cyclones-a-la-reunion.html), i.e. during the compilation of observational data in [46]. However, no impacts on *Culicoides* populations or significant discrepancies with predictions were noted. Moreover, it should be stressed that NRMSE tends to be higher for low abundances even if the order of magnitude of observations and predictions is comparable, as it is the case for *C.* *grahamii* (maximum catch: 28 individuals per trap; maximum predicted abundance: 52 individuals per trap).

A spatial autocorrelation analysis to identify the lack of independence between farms was not provided in the modeling process. The median distance (range in brackets) between farms is 1.775 (0.22–7.52) km. Considering the low dispersal capacities of the *Culicoides* [38, 47, 81], most farms sampled in March 2018 can be considered sufficiently isolated so that the *Culicoides* population on one farm did not significantly affect those in the neighborhood. However, it is normal that farms close to each other, and therefore subject to similar climatic and environmental conditions, have similar *Culicoides* compositions. The spatial inspection of the residuals (Fig. 6) did not show obvious signs of autocorrelation except for an overestimation cluster of *C. imicola* in the northeastern highland and an underestimation cluster of *C. kibatiensis* abundances in the western highland. Apart from these two clusters, residuals were not spatially autocorrelated, indicating that the inclusion of numerous climatic and environmental explanatory variables in the models could have been sufficient to take into account any spatial autocorrelation of *Culicoides* abundance.

For *C. imicola*, model estimates of abundance were satisfactory only if outliers were not considered. *Culicoides imicola* abundance was overestimated in the municipality of Salazie and in some sites on coastal areas (Fig. 6). These sites are associated with a high percentage of bare rock, a variable originally associated with high *C. imicola* abundance. Because of the exponential function, the *C. imicola* abundance model is very sensitive to this variable, which can lead to high volatility in the predictions. This tends to have a strong impact on the NRMSE. In addition, the municipality of Salazie also has specific features. As a geological cirque, this municipality is a very isolated region of the island, surrounded by cliffs of several hundred meters, with very rugged relief and with stony and very gullied soils [82]. In this area, the climatic and environmental conditions considered in our models were very favorable for high *Culicoides* abundances, but its particular landscape and soil characteristics, which were not exploited in the models, may explain the lower abundances observed in March 2018. Indeed, if landscape can play a role in local *Culicoides* abundance [39, 83, 84], the type of soil may limit *Culicoides* development, as in a *C.* *imicola*-free zone in South Africa, where the soil is sandy, poor in nutrients and too well drained to sustain *Culicoides* larvae [85].

Another source of differences between predictions and the observations of March 2018 could come from the variable estimates. For example, the building opening size was estimated according to the general configuration of the type of production and not from field observations as in [46]. Greater distances to weather stations could lead to approximates of climate data given the diversity of the island's microclimates.

Finally, the type of animal hosts in the vicinity of the traps could also be a source of variation. By construction, the predictions from the temporal dynamics model built in Grimaud et al. [46] reflect the expected catches in the vicinity of cattle. The farms selected for the March 2018 campaign were mainly cattle farms (82 out of 100, Additional file 2) and therefore correspond to the conditions under which the temporal models were constructed in Grimaud et al. [46]. However, considering the predictions to all husbandry locations in this study, other animal types, such as sheep, goats, deer and horses, were included, assuming an equivalent importance of the host type on the composition of *Culicoides* species. However, some *Culicoides* species may be associated with one host type more than another by their host preference or the behavior [47, 48, 86, 87]. Unfortunately, no comparative study was carried out in Reunion Island to consider the differences induced by the type of host in the vicinity of the traps and therefore requires careful consideration of predictions.

Usually, for *Culicoides*, the spatial and temporal components of their abundance distribution are modeled separately. Only Brugger and Ruble [41] and Rigot et al. [43] built spatio-temporal models on *C. obsoletus* spp. and *C. imicola*, respectively. However, these models did not incorporate environmental variables such as land use, eco-climate, host type and density although Conte et al. [88] and Purse et al. [83] suggested the increased model accuracy when estimating *Culicoides* abundance. In this study, environmental and host variables as well as the entrance size of buildings, which may influence *Culicoides* abundance depending on their exophilic or endophilic behaviors, were included for the first time to model the spatio-temporal distribution of *Culicoides*. It should be noted that the use of the Ocelet modeling platform, a free software dedicated to the modeling of spatial dynamics, greatly facilitated the integration and processing of the geographic information corresponding to the climate, environment and host variables [66]. In particular, its capacity to facilitate the formalization of the link between these data and entities, the interactions between entities and the definition of a scenario were proved to be extremely useful.

Recently, spatio-temporal models of arthropod vectors have sparked the interest of public health authorities in Reunion Island and its close sister island, Mauritius. Indeed, a similar model was developed for *Aedes albopictus*, a vector of dengue and chikungunya viruses in the Indian Ocean, and transferred to local authorities [89] to help prioritize action areas where public awareness and vector control measures should be implemented. For *Culicoides*, control methods are not as developed as for mosquitoes [90], limiting, for the moment, the development of an applied tool used by pest management units. The development of control actions is further challenged by the fact that our results suggest that vectors are present in nearly every farm in Reunion Island and during sufficiently long periods of time to support BTV and EHDV transmission. Yet, a tool equivalent to that developed for mosquitoes in Reunion Island would help raise awareness, train and support decision-making relative to prevention strategies and enable testing control methods. In addition, for horses that may develop summer dermatitis because of *Culicoides* bites, such a tool could be useful to anticipate when and where protective measures for these animals should be implemented.

## Conclusions

The modeling approach enabled developing dynamic maps of abundance for the five *Culicoides* species present in Reunion Island for the first time to our knowledge. Indeed, the use of the GIS and Ocelet spatial modeling platform enabled easy integration of different climatic and environmental variables and facilitated the development of dynamic risk maps. As the approach relied on the extrapolation to the entire island of dynamic models initially developed in 11 sites, an extensive trapping campaign was set up to validate models on a new dataset. Models suggest that the five species have a wide distribution and long period of activity, which could support continuous circulation of BTV and EHDV. Fit of abundance models were acceptable for *C. bolitinos, C. enderleini and C. kibatiensis* but were not as good for *C. imicola* and *C. grahamii*. Inclusion of other parameters such as soil type or host factors may contribute to improving model fit. To better assess BTV and EDHV transmission risk, it now seems crucial to evaluate the vector competence of each species. Then, abundance and competence estimates should be integrated to build more complete disease transmission models. There are currently no effective methods to control BT or EHD in Reunion Island, either against the virus (vaccination—as there are too many serotypes circulating on the island) or against *Culicoides* (vector control). More effort is thus needed to develop innovative prevention and/or control methods applicable to the Reunionese context. In any case, given the wide distribution and overlapping periods of activity of the five species of *Culicoides*, disease control strategies will probably need to be integrated at both the level of the breeder (for example, to determine the period of control on the farm) and the level of the health authorities (to determine priority interventions and target areas). Awaiting effective control measures, these first results should be used to communicate and raise awareness among animal health actors and breeders in Reunion Island on BTV and EHDV vector spatial dynamics.

## Supplementary Information

**Additional file 1: Table S1.** Model parameters for *C. bolitinos*. **Table S2.** Model parameters for *C. enderleini*. **Table S3.** Model parameters for *C. grahamii*. **Table S4.** Model parameters for *C. imicola*. **Table S5.** Model parameters for *C. kibatiensis*.

**Additional file 2: Table S6. **Observed *Culicoides* abundance during the sampling campaign from 7 to 22 March 2018.

**Additional file 3. **Movie of the spatio-temporal distribution of *C. bolitinos*.

**Additional file 4. **Movie of the spatio-temporal distribution of *C. enderleini*.

**Additional file 5. **Movie of the spatio-temporal distribution of *C. grahamii*.

**Additional file 6. **Movie of the spatio-temporal distribution of *C. imicola*.

**Additional file 7. **Movie of the spatio-temporal distribution of *C. kibatiensis*.

## Data Availability

All sample are available upon request to the corresponding authors. Ocelet codes are available on Cirad Dataverse.

## Abbreviations

AHS: African horse sickness
AUC: Area under curve
BT(V): Bluetongue (virus)
DAUPI: Urban planning and native plants approach
EHD(V): Epizootic hemorrhagic disease (virus)
GIS: Geographic information system
GDS: Groupement de défense sanitaire
MODIS: Moderate resolution imaging spectroradiometer
NDVI: Normalized difference vegetation index
(N)RMSE: (Normalized) root mean square error
OVI: Onderstepoort Veterinary Institute
ROC: Receiver-operating characteristic
ROC AUCs: Area under the ROC curve
VBDs: Vector-borne diseases

## References

[1] Reisen WK (2010). Landscape epidemiology of vector-borne diseases. Annu Rev Entomol.

[2] Lambin EF, Tran A, Vanwambeke SO, Linard C, Soti V (2010). Pathogenic landscapes: interactions between land, people, disease vectors, and their animal hosts. Int J Health Geogr.

[3] Moore CG (2008). Interdisciplinary research in the ecology of vector-borne diseases: opportunities and needs. J Vector Ecol.

[4] Robert V. Introduction à l'entomologie médicale et vétérinaire. In: Duvallet G, Fontenille D, Robert V, editors. Entomologie médicale et vétérinaire, Quae & IRD edn; 2017. p. 37–59.

[5] Kilpatrick AM, Randolph SE (2012). Drivers, dynamics, and control of emerging vector-borne zoonotic diseases. Lancet.

[6] Hartemink N, Vanwambeke SO, Purse BV, Gilbert M, Van Dyck H (2015). Towards a resource-based habitat approach for spatial modelling of vector-borne disease risks. Biol Rev.

[7] Gage KL, Burkot TR, Eisen RJ, Hayes EB (2008). Climate and vectorborne diseases. Am J Prev Med.

[8] Kluiters G, Sugden D, Guis H, McIntyre KM, Labuschagne K, Vilar MJ (2013). Modelling the spatial distribution of *Culicoides* biting midges at the local scale. J Appl Ecol.

[9] Eisen L, Eisen RJ (2011). Using geographic information systems and decision support systems for the prediction, prevention, and control of vector-borne diseases. Annu Rev Entomol.

[10] Rogers DJ, Randolph SE (2003). Studying the global distribution of infectious diseases using GIS and RS. Nat Rev Microbiol.

[11] Purse B, Carpenter S, Venter G, Bellis G, Mullens B (2015). Bionomics of temperate and tropical *Culicoides* midges: knowledge gaps and consequences for transmission of *Culicoides*-borne viruses. Annu Rev Entomol.

[12] Mellor P, Boorman J, Baylis M (2000). *Culicoides* biting midges: their role as arbovirus vectors. Annu Rev Entomol.

[13] Capela R, Purse B, Pena I, Wittman E, Margarita Y, Capela M (2003). Spatial distribution of *Culicoides* species in Portugal in relation to the transmission of African horse sickness and bluetongue viruses. Med Vet Entomol.

[14] William W, Bülent A, Thomas B, Eduardo B, Marieta B, Olivier B (2018). The importance of vector abundance and seasonality: Results from an expert consultation VectorNet project. EFSA Supporting Publications.

[15] Mellor P, Wittmann E (2002). Bluetongue virus in the Mediterranean Basin 1998–2001. Vet J.

[16] Carpenter S, Wilson A, Mellor PS (2009). *Culicoides* and the emergence of bluetongue virus in northern Europe. Trends Microbiol.

[17] Calistri P, Goffredo M, Caporale V, Meiswinkel R (2003). The distribution of *Culicoides imicola* in Italy: application and evaluation of current Mediterranean models based on climate. J Vet Med B Infect Dis Vet Public Health.

[18] Savini G, Afonso A, Mellor P, Aradaib I, Yadin H, Sanaa M (2011). Epizootic heamorragic disease. Res Vet Sci.

[19] Maclachlan NJ, Zientara S, Savini G, Daniels P (2015). Epizootic haemorrhagic disease. Rev Sci Tech.

[20] MacLachlan NJ, Guthrie AJ (2010). Re-emergence of bluetongue, African horse sickness, and other orbivirus diseases. Vet Res.

[21] Diarra M, Fall M, Fall AG, Diop A, Seck MT, Garros C (2014). Seasonal dynamics of *Culicoides* (Diptera: Ceratopogonidae) biting midges, potential vectors of African horse sickness and bluetongue viruses in the Niayes area of Senegal. Parasite Vector.

[22] Purse B, Tatem A, Caracappa S, Rogers D, Mellor P, Baylis M (2004). Modelling the distributions of *Culicoides* bluetongue virus vectors in Sicily in relation to satellite-derived climate variables. Med Vet Entomol.

[23] Torina A, Caracappa S, Mellor P, Baylis M, Purse B (2004). Spatial distribution of bluetongue virus and its *Culicoides* vectors in Sicily. Med Vet Entomol.

[24] Baylis M, Meiswinkel R, Venter G (1999). A preliminary attempt to use climate data and satellite imagery to model the abundance and distribution of *Culicoides imicola* (Diptera: Ceratopogonidae) in southern Africa. J S Afr Vet Assoc.

[25] Baylis M, Mellor P, Wittmann E, Rogers D (2001). Prediction of areas around the Mediterranean at risk of bluetongue by modelling the distribution of its vector using satellite imaging. Vet Rec.

[26] Baylis M, O’Connell L, Purse B (2004). Modelling the distribution of bluetongue vectors. Vet Ital.

[27] Baylis M, Rawlings P (1998). Modelling the distribution and abundance of *Culicoides imicola* in Morocco and Iberia using climatic data and satellite imagery. Arch Virol Suppl.

[28] Tatem A, Baylis M, Mellor P, Purse B, Capela R, Pena I (2003). Prediction of bluetongue vector distribution in Europe and North Africa using satellite imagery. Vet Microbiol.

[29] Calvete C, Estrada R, Miranda M, Borras D, Calvo J, Lucientes J (2008). Modelling the distributions and spatial coincidence of bluetongue vectors *Culicoides imicola* and the *Culicoides obsoletus* group throughout the Iberian Peninsula. Med Vet Entomol.

[30] Diarra M, Fall M, Fall AG, Diop A, Lancelot R, Seck MT (2018). Spatial distribution modelling of *Culicoides* (Diptera: Ceratopogonidae) biting midges, potential vectors of African horse sickness and bluetongue viruses in Senegal. Parasite Vector.

[31] Guichard S, Guis H, Tran A, Garros C, Balenghien T, Kriticos DJ (2014). Worldwide niche and future potential distribution of *Culicoides imicola*, a major vector of bluetongue and African horse sickness viruses. PLoS ONE.

[32] Federici V, Ippoliti C, Goffredo M, Catalani M, Di Provvido A, Santilli A (2016). Epizootic haemorrhagic disease in Italy: vector competence of indigenous Culicoides species and spatial multicriteria evaluation of vulnerability. Vet Ital.

[33] Cuéllar AC, Kjær LJ, Baum A, Stockmarr A, Skovgard H, Nielsen SA (2020). Modelling the monthly abundance of *Culicoides* biting midges in nine European countries using Random Forests machine learning. Parasite Vector.

[34] Blanda V, Blanda M, La Russa F, Scimeca R, Scimeca S, D’Agostino R (2018). Geo-statistical analysis of Culicoides spp distribution and abundance in Sicily, Italy. Parasite Vector..

[35] Sánchez-Matamoros A, Sánchez-Vizcaíno J, Rodríguez-Prieto V, Iglesias E, Martínez-López B (2016). Identification of suitable areas for African horse sickness virus infections in Spanish equine populations. Transbound Emerg Dis.

[36] Cuéllar AC, Kjær LJ, Kirkeby C, Skovgard H, Nielsen SA, Stockmarr A (2018). Spatial and temporal variation in the abundance of *Culicoides* biting midges (Diptera: Ceratopogonidae) in nine European countries. Parasite Vector.

[37] Villard P, Muñoz F, Balenghien T, Baldet T, Lancelot R, Hénaux V (2019). Modeling *Culicoides* abundance in mainland France: implications for surveillance. Parasite Vector.

[38] Kirkeby C, Bødker R, Stockmarr A, Lind P (2013). Spatial abundance and clustering of *Culicoides* (Diptera: Ceratopogonidae) on a local scale. Parasite Vector.

[39] Guis H: Géomatique et épidémiologie: caractérisation des paysages favorables à *Culicoides imicola*, vecteur de la fièvre catarrhale ovine en Corse. Université de Franche-Comté; 2007.

[40] Quaglia AI, Blosser EM, McGregor BL, Runkel AE, Sloyer KE, Erram D (2020). Tracking community timing: pattern and determinants of seasonality in *Culicoides* (Diptera: Ceratopogonidae) in Northern Florida. Viruses.

[41] Brugger K, Rubel F (2013). Bluetongue disease risk assessment based on observed and projected *Culicoides obsoletus* spp. vector densities. PLoS ONE.

[42] Hartemink N, Purse B, Meiswinkel R, Brown HE, De Koeijer A, Elbers A (2009). Mapping the basic reproduction number (R0) for vector-borne diseases: a case study on bluetongue virus. Epidemics.

[43] Rigot T, Conte A, Goffredo M, Ducheyne E, Hendrickx G, Gilbert M (2012). Predicting the spatio-temporal distribution of *Culicoides imicola* in Sardinia using a discrete-time population model. Parasite Vector.

[44] Ensoy C, Aerts M, Welby S, Van der Stede Y, Faes C (2013). A dynamic spatio-temporal model to investigate the effect of cattle movements on the spread of bluetongue BTV-8 in Belgium. PLoS ONE.

[45] Desvars A, Grimaud Y, Guis H, Esnault O, Allene X, Gardes L (2015). First overview of the *Culicoides* Latreille (Diptera: Ceratopogonidae) livestock associated species of Reunion Island. Indian Ocean Acta Trop.

[46] Grimaud Y, Guis H, Chiroleu F, Boucher F, Tran A, Rakotoarivony I (2019). Modelling temporal dynamics of *Culicoides* Latreille (Diptera: Ceratopogonidae) populations on Reunion Island (Indian Ocean), vectors of viruses of veterinary importance. Parasite Vector.

[47] Bakhoum MT, Fall M, Seck M, Gardes L, Fall A, Diop M (2016). Foraging range of arthropods with veterinary interest: new insights for Afrotropical *Culicoides* biting midges (Diptera: Ceratopogonidae) using the ring method. Acta Trop.

[48] Fall M, Fall AG, Seck MT, Bouyer J, Diarra M, Lancelot R (2015). Host preferences and circadian rhythm of *Culicoides* (Diptera: Ceratopogonidae), vectors of African horse sickness and bluetongue viruses in Senegal. Acta Trop.

[49] Venter G, Meiswinkel R, Nevill E, Edwardes M (1996). *Culicoides* (Diptera: Ceratopogonidae) associated with livestock in the Onderstepoort area, Gauteng, South Africa as determined by light-trap collections. Onderstepoort J Vet Res.

[50] Walker A, Boreham P (1976). Blood feeding of *Culicoides* (Diptera, Ceratopogonidae) in Kenya in relation to the epidemiology of bluetongue and ephemeral fever. Bull entomol Res.

[51] Goffredo M, Savini G, Quaglia M, Molini U, Federici V, Catalani M (2015). Orbivirus detection from *Culicoides* collected on African horse sickness outbreaks in Namibia. Vet Ital.

[52] Venter G, Mellor P, Paweska J (2006). Oral susceptibility of South African stock-associated Culicoides species to bluetongue virus. Med Vet Entomol.

[53] Gerdes G (2004). A South African overview of the virus, vectors, surveillance and unique features of bluetongue. Vet Ital.

[54] Paweska J, Venter G, Mellor P (2002). Vector competence of South African Culicoides species for bluetongue virus serotype 1 (BTV-1) with special reference to the effect of temperature on the rate of virus replication in *C. imicola* and *C. bolitinos*. Med Vet Entomol..

[55] Paweska J, Venter G, Hamblin C (2005). A comparison of the susceptibility of *Culicoides imicola* and *C. bolitinos* to oral infection with eight serotypes of epizootic haemorrhagic disease virus. Med Vet Entomol..

[56] WHO. Manual on practical entomology in malaria. Part I. Geneva: World Health Organisation Division of Malaria and Other Parasitic Diseases; 1975.

[57] Barré N, Eramus B, Gautier A, Reme A, Valin R. La bluetongue, nouvelle maladie des ovins à la Réunion (Océan Indien). Rev Elev Med Vet Pay. 1985;38. 3018871

[58] Bréard E, Sailleau C, Gourreau J (2004). Outbreak of epizootic haemorrhagic disease on the island of Réunion. Vet Rec.

[59] Cêtre-Sossah C, Roger M, Sailleau C, Rieau L, Zientara S, Bréard E (2014). Epizootic haemorrhagic disease virus in Reunion Island: evidence for the circulation of a new serotype and associated risk factors. Vet Microbiol.

[60] Raunet M. Le milieu physique et les sols de l'île de la Réunion. Conséquences pour la mise en valeur agricole. Montpellier: Cirad; 1991.

[61] Didan K. MOD13Q1 MODIS/Terra vegetation indices 16-day L3 global 250m SIN grid V006. NASA EOSDIS Land Processes DAAC. 2015.

[62] Londoño Villegas MM: Développement d'une méthodologie pour la construction de cartes d'occupation du sol de l'île de la Réunion. Université Paul Valéry; 2017.

[63] Dupuy S, Gaetano R, 2019. La Réunion - Carte d'occupation du sol, 2017 (Spot6/7). CIRAD Dataverse, V1. 10.18167/DVN1/OMHZQL.

[64] Zeileis A, Grothendieck G (2005). zoo: S3 infrastructure for regular and irregular time series. J Stat Softw.

[65] QGIS Development Team. QGIS Geographic Information System. Open Source Geospatial Foundation Project. http://qgisosgeo.org. 2015.

[66] Degenne P, Lo Seen D (2016). Ocelet: simulating processes of landscape changes using interaction graphs. SoftwareX.

[67] Chopart J-L, Mézino M, Le Mezo L (2002). Relations entre l'altitude et la température mensuelle de l'air dans l'ouest de la Réunion. Rev Agri Sucr Maurice.

[68] Gnauck A (2004). Interpolation and approximation of water quality time series and process identification. Anal Bioanal Chem.

[69] Park SH, Goo JM, Jo C-H (2004). Receiver operating characteristic (ROC) curve: practical review for radiologists. Korean J Radiol.

[70] R Core Team. R: A language and environment for statistical computing. Vienna: R Foundation for Statistical Computing; 2017. https://www.R-project.org/.

[71] Robin X, Turck N, Hainard A, Tiberti N, Lisacek F, Sanchez J-C, et al: Package ‘pROC’. 2019. 10.1186/1471-2105-12-77 PMC3068975 21414208

[72] Zambrano-Bigiarini M. Package ‘hydroGOF’. Goodness-of-fit Functions for Comparison of Simulated and Observed. 2017.

[73] Donnelly MJ, Simard F, Lehmann T (2002). Evolutionary studies of malaria vectors. Trends Parasitol.

[74] Meiswinkel R, Paweska J (2003). Evidence for a new field *Culicoides* vector of African horse sickness in South Africa. Prev Vet Med.

[75] Sanders CJ, Shortall CR, England M, Harrington R, Purse B, Burgin L (2019). Long-term shifts in the seasonal abundance of adult *Culicoides* biting midges and their impact on potential arbovirus outbreaks. J Appl Ecol.

[76] Baylis M, Mellor PS, Meiswinkel R (1999). Horse sickness and ENSO in South Africa. Nature.

[77] Johansson MA, Cummings DA, Glass GE (2009). Multiyear climate variability and dengue—El Nino southern oscillation, weather, and dengue incidence in Puerto Rico, Mexico, and Thailand: a longitudinal data analysis. PLoS Med..

[78] Poveda G, Graham NE, Epstein PR, Rojas W, Quiñones ML, Velez ID, et al. Climate and ENSO variability associated with vector-borne diseases in Colombia. El Niño and the southern oscillation, Multiscale variability and global and regional impacts. 2000;1:183–204.

[79] Linthicum KJ, Anyamba A, Tucker CJ, Kelley PW, Myers MF, Peters CJ (1999). Climate and satellite indicators to forecast Rift Valley fever epidemics in Kenya. Science.

[80] Guis H, Caminade C, Calvete C, Morse AP, Tran A, Baylis M (2011). Modelling the effects of past and future climate on the risk of bluetongue emergence in Europe. J R Soc Interface.

[81] Kluiters G, Swales H, Baylis M (2015). Local dispersal of palaearctic *Culicoides* biting midges estimated by mark-release-recapture. Parasite Vector.

[82] Raunet M. milieu physique et les sols de l'ile de la Réunion. 1991.

[83] Purse B, Falconer D, Sullivan M, Carpenter S, Mellor P, Piertney S (2012). Impacts of climate, host and landscape factors on *Culicoides* species in Scotland. Med Vet Entomol.

[84] Ippoliti C, Gilbert M, Vanhuysse S, Goffredo M, Satta G, Wolff E (2013). Can landscape metrics help determine the *Culicoides imicola* distribution in Italy?. Geospat Health.

[85] Meiswinkel R (1997). Discovery of a *Culicoides imicola*-free zone in South Africa: preliminary notes and potential significance. Onderstepoort J Vet Res.

[86] Meiswinkel R, Labuschagne K, Baylis M, Mellor P (2004). Multiple vectors and their differing ecologies: observations on two bluetongue and African horse sickness vector *Culicoides* species in South Africa. Vet Ital.

[87] Viennet E, Garros C, Lancelot R, Allène X, Gardès L, Rakotoarivony I (2011). Assessment of vector/host contact: comparison of animal-baited traps and UV-light/suction trap for collecting *Culicoides* biting midges (Diptera: Ceratopogonidae), vectors of Orbiviruses. Parasite Vector.

[88] Conte A, Goffredo M, Ippoliti C, Meiswinkel R (2007). Influence of biotic and abiotic factors on the distribution and abundance of *Culicoides imicola* and the Obsoletus Complex in Italy. Vet Parasitol.

[89] Tran A, Mangeas M, Demarchi M, Roux E, Degenne P, Haramboure M (2020). Complementarity of empirical and process-based approaches to modelling mosquito population dynamics with Aedes albopictus as an example—application to the development of an operational mapping tool of vector populations. PLoS ONE.

[90] Harrup LE, Miranda MA, Carpenter S (2016). Advances in control techniques for *Culicoides* and future prospects. Vet Ital.

