# Supplementary material for: Spatio-temporal modelling of Culicoides Latreille (Diptera: Ceratopogonidae) populations on Reunion Island (Indian Ocean)
Source: Parasit Vectors. 2021 May 27;14:288. doi: 10.1186/s13071-021-04780-9 (PMC8161615; doi:10.1186/s13071-021-04780-9)
Supplement: Supplementary file 1 — Additional file 1: Table S1. Model parameters for C. bolitinos. Table S2. Model parameters for C. enderleini. Table S3. Model parameters for C. grahamii. Table S4. Model parameters for C. imicola. Table S5. Model parameters for C. kibatiensis. [file 13071_2021_4780_MOESM1_ESM.docx]

**Table S1: model parameters for *C. bolitinos***

| **Presence estimation (threshold** ^a^ **= 0.555)** | | | | **Abundance estimation** | | |
| --- | --- | --- | --- | --- | --- | --- |
| Variables | Classes | Coeff. ^b^ | | Variables | Classes | Coeff. |
| *Intercept* | | -12.175 | | *Intercept* |  | 1.096 |
| Maximum temperature during the DoS ^c^ (°C) | | 0.120 | | Log10 minimum temperature 10 days before the DoS (°C) | | -4.654 |
| Maximum temperature 14 days before DoS (°C) | | 0.210 | | Maximum temperature 35 days before the DoS (°C) | | 0.192 |
| Average rain from day 24 to day 21 before DoS (mm) | | -0.067 | | Log10 rain during the DoS (mm) | | 0.297 |
| NDVI 18 days before DoS | | 0.029 | | Rain 9 days before DoS (mm) | | 0.015 |
| % of land use by planted forest in 0.5 km radius (%; max. ^d^ 0.6%) | | | -3.513 | Average log10 rain from day 25 to day 23 before DoS (mm) | | 0.804 |
| Number of small ruminants in 0.5 km radius (max. 80) | | -0.044 | | % of land use by savannah in 0.5 km radius (%) | | -0.050 |
| Number of farms in 2 km radius (max. 69) | | 0.075 | | % of land use by planted forest in 1 km radius (%; max. 4.7%) | | -0.554 |
| Minimum temperature during the 14 days before DoS (°C) | (7.04, 13.7] | 2.061 | | Number of cattle in 1 km radius (max. 884) | | 0.003 |
|  | (13.7, 16.9] | 1.026 | | Rain 1 day before DoS (mm) | (0.2, 1.5] | -0.239 |
|  | > 16.9 | 0.634 | |  | (1.5, 6.92] | 0.109 |
|  |  |  | |  | > 6.92 | 0.636 |
|  |  |  | | Global radiation 1 day before DoS (kJ.cm^-2^) | (1.09, 1.53] | 0.415 |
|  |  |  | |  | (1.53, 1.97] | 0.669 |
|  |  |  | |  | > 1.97 | -0.057 |
|  |  |  | | Global radiation during the DoS (kJ.cm^-2^) | (1.25, 1.8] | 0.527 |
|  |  |  | |  | > 1.8 | 0.851 |
|  |  |  | | Building opening size (%) | (25, 100] | 1.403 |
|  |  |  | |  | Enclosure | 0.766 |

^a^ the threshold defining a positive abundance; ^b^ Coeff.: coefficient; ^c^ DoS: day of simulation; ^d^ max.: the maximum limit value for which the estimate of presence and/or abundance can be made.

**Table S2: model parameters for *C. enderleini***

| **Presence estimation (threshold** ^a^ **= 0.2)** | | | **Abundance estimation** | | |
| --- | --- | --- | --- | --- | --- |
| Variables | Classes | Coeff. ^b^ | Variables | Classes | Coeff. |
| *Intercept* | | -3.666 | *Intercept* |  | -10.487 |
| Maximum temperature during the DoS ^c^ (°C) | | 0.246 | Log10 minimum temperature during the 14 days before DoS (°C) | | 4.719 |
| Maximum temperature during the 14 days before DoS (°C) | | 0.338 | Log10 rain 9 days before DoS (mm) | | 1.126 |
| Average humidity from day 27 to day 17 before DoS (%) | | -0.080 | Number of small ruminants in 1 km radius (max. ^d^ 65) | | 0.070 |
| Average humidity from day 41 to day 35 before DoS (%) | | -0.126 | Number of total animals in 1 km radius (max. 271) | | 0.022 |
| Average rain from day 48 to day 44 before DoS (mm) | | 0.062 | Average wind during the DoS (m.s^-1^) | (0.512, 0.9] | -0.491 |
| Average wind 1 day before DoS (m.s^-1^) | | -1.209 |  | (0.9, 1.2] | -0.281 |
| NDVI | | 0.051 |  | (1.2, 1.46] | 1.713 |
| % land use by forest in 2 km radius (%) | | -0.045 |  | > 1.46 | -1.982 |
| % land use by orchard in 2 km radius (%) | | -0.088 | Eco-climatic area | Lwf ^e^ | 1.157 |
| Rain during the DoS (mm) | (0.1, 1.1] | 1.164 |  | Mrf & Tf ^f^ | -3.401 |
|  | (1.1, 6.3] | -0.415 |  |  |  |
|  | > 6.3 | -0.740 |  |  |  |

^a^ the threshold defining a positive abundance; ^b^ Coeff.: coefficient; ^c^ DoS: day of simulation; ^d^ max.: the maximum limit value for which the estimate of presence and/or abundance can be made; ^e^ Lwf: lowland wet forest; ^f^ Mrf & Tf: mountain rain forest and tamarind forest.

**Table S3: model parameters for *C. grahamii***

| **Presence estimation (threshold** ^a^ **= 0.27)** | | | **Abundance estimation** | | |
| --- | --- | --- | --- | --- | --- |
| Variables | Classes | Coeff. ^b^ | Variables | Classes | Coeff. |
| *Intercept* | | -15.818 | *Intercept* |  | 1.765 |
| Average humidity from day 20 to day 15 before DoS ^c^ (%) | | 0.049 | NDVI 21 days before DoS | | -0.020 |
| Average humidity from day 48 to day 43 before DoS (%) | | 0.064 | Rain 1 day before DoS (mm) | (0.2, 1.5] | 0.936 |
| Log10 Average rain from day 3 to day 2 before DoS (mm) | | -0.723 |  | (1.5, 6.92] | 1.489 |
| Average rain from day 29 to day 24 before DoS (mm) | | -0.042 |  | > 6.92 | 1.472 |
| Rain 48 days before DoS (mm) | | 0.036 | Rain during the DoS (mm) | (0.1, 1.1] | -0.864 |
| Average wind 1 day before DoS (m.s^-1^) | | -0.599 |  | (1.1, 6.3] | -1.899 |
| NDVI | | 0.022 |  | > 6.3 | -0.528 |
| NDVI 31 days before DoS | | 0.043 | Global radiation 1 day before DoS (kJ.cm^-2^) | (1.01, 1.38] | -0.333 |
| Number of deer and horses in 2 km radius (max. ^d^ 52) | | 0.034 |  | (1.38, 1.68] | -0.134 |
| Minimum temperature during the DoS (°C) | (12.1, 15.1] | 1.213 |  | (1.68, 2.1] | 1.249 |
|  | (15.1, 18] | 0.370 |  | > 2.1 | -1.267 |
|  | (18, 21] | 0.624 | Eco-climatic area | Lwf | -0.301 |
|  | > 21 | 0.411 |  | Wcmrf | 1.990 |
| Eco-climatic area | Lwf ^e^ | 0.447 |  | Lcmrf | 1.340 |
|  | Wcmrf ^f^ | 0.311 | Building opening size (%) | (25, 100] | -2.009 |
|  | Lcmrf ^g^ | 2.648 |  | Enclosure | -0.732 |

^a^ the threshold defining a positive abundance; ^b^ Coeff.: coefficient; ^c^ DoS: day of simulation; ^d^ max.: the maximum limit value for which the estimate of presence and/or abundance can be made; ^e^ Lwf: lowland wet forest; ^f^ Wcmrf: windward coast mountain rain forest; ^g^ Lwmrf: leeward coast mountain rain forest.

**Table S4: model parameters for *C. imicola***

| **Presence estimation (threshold** ^a^ **= 0.61)** | | | **Abundance estimation** | | |
| --- | --- | --- | --- | --- | --- |
| Variables | Classes | Coeff. ^b^ | Variables | Classes | Coeff. |
| *Intercept* | | -9.855 | *Intercept* |  | -2.718 |
| Maximum temperature during the DoS ^c^ (°C) | | 0.160 | Maximum temperature during the DoS (°C) | | 0.190 |
| Maximum temperature during the 14 days before DoS (°C) | | 0.214 | Maximum temperature during the 14 days before DoS (°C) | | 0.090 |
| Rain during the DoS (mm) | | 0.028 | Humidity at day 26 before DoS (%) | | -0.027 |
| Rain 4 days before DoS (mm) | | -0.033 | Rain during the DoS (mm) | | -0.013 |
| Average wind 1 day before DoS (m.s^-1^) | | -0.477 | NDVI at day 50 before DoS | | -0.020 |
| % land use by urban area in 2 km radius (%) | | 0.052 | % land use by planted forest in 1 km radius (%; max. 4.7%)) | | -0.735 |
| Number of small ruminants in 1 km radius (max. ^d^ 246) | | -0.010 | % land use by bare rock in 2 km radius (%; max. 1.6%) | | 4.195 |
| Building opening size (%) | (25, 100] & | 2.746 | Number of deer and horses in 2 km radius (max. 52) | | -0.031 |
|  | enclosure |  | Rain 1 day before DoS (mm) | (0.2, 1.5] | -0.815 |
|  |  |  |  | (1.5, 6.92] | 0.037 |
|  |  |  |  | > 6.92 | -0.332 |
|  |  |  | NDVI | (60, 67] | 0.440 |
|  |  |  |  | (67, 73] | 0.165 |
|  |  |  |  | (73, 78] | -0.441 |
|  |  |  |  | > 78 | 0.527 |
|  |  |  | Eco-climatic area | Df & sav. ^e^ | 1.524 |
|  |  |  |  | Wcmrf ^f^ | 4.621 |
|  |  |  |  | Lcmrf & Tf ^g^ | 1.368 |

^a^ the threshold defining a positive abundance; ^b^ Coeff.: coefficient; ^c^ DoS: day of simulation; ^d^ max.: the maximum limit value for which the estimate of presence and/or abundance can be made; ^e^ Df & sav.: dry forest and savannah; ^f^ Wcmrf: windward coast mountain rain forest; ^g^ Lwmrf & Tf: leeward coast mountain rain forest and tamarind forest.

**Table S5: model parameters for *C. kibatiensis***

| **Presence estimation (threshold** ^a^ **= 0.58)** | | | **Abundance estimation** | | |
| --- | --- | --- | --- | --- | --- |
| Variables | Classes | Coeff. ^b^ | Variables | Classes | Coeff. |
| *Intercept* | | 6.984 | *Intercept* |  | 3.305 |
| Maximum temperature during the DoS ^c^ (°c) | | -0.095 | Minimum temperature during the 14 days before DoS (°C) | | -0.093 |
| Maximum temperature during the 14 days before DoS (°C) | | -0.369 | Maximum temperature 44 days before DoS (°C) | | -0.077 |
| Average humidity from day 50 to day 3 before DoS (%) | | 0.053 | Global radiation during the DoS (kJ.cm^-2^) | | -0.304 |
| Log10 average rain from day 11 to day 10 before DoS (mm) | | 0.747 | Number of deer and horses in 0.5 km radius (max. 12) | | 0.141 |
| NDVI 29 days before DoS | | 0.038 | Number of deer and horses in 2 km radius (max. 52) | | 0.032 |
| % land use by bare rock in 2 km radius (%; max. ^d^ 1.6%) | | -1.504 | Minimum temperature during the DoS (°C) | (12.1, 15.1] | 0.454 |
| Number of deer and horses in 2 km radius (max. 52) | | 0.038 |  | (15.1, 18] | -0.070 |
| Eco-climatic area | Lwf ^e^ | -1.958 |  | (18, 21] | -0.427 |
|  | Wcmrf ^f^ | -0.907 |  | > 21 | 0.780 |
|  | Lcmrf ^g^ | -0.544 | Rain 1 day before DoS (mm) | (0.2, 1.5] | 0.5995 |
|  | Tf ^h^ | -2.465 |  | (1.5, 6.92] | 0.987 |
|  |  |  |  | > 6.92 | 0.706 |
|  |  |  | Eco-climatic area | Lwf | 1.610 |
|  |  |  |  | Wcmrf | 3.182 |
|  |  |  |  | Lcmrf | 1.691 |
|  |  |  |  | Tf | -0.125 |

^a^ the threshold defining a positive abundance; ^b^ Coeff.: coefficient; ^c^ DoS: day of simulation; ^d^ max.: the maximum limit value for which the estimate of presence and/or abundance can be made; ^e^ Lwf.: lawland wet forest; ^f^ Wcmrf: windward coast mountain rain forest; ^g^ Lwmrf: leeward coast mountain rain forest; ^h^ Tf: tamarind forest.
