# Supplementary material for: Spatio-temporal modelling of Culicoides Latreille (Diptera: Ceratopogonidae) populations on Reunion Island (Indian Ocean)
Source: Parasit Vectors. 2021 May 27;14:288. doi: 10.1186/s13071-021-04780-9 (PMC8161615; doi:10.1186/s13071-021-04780-9)
Supplement: Supplementary file 2 — Additional file 2: Table S6. Observed Culicoides abundance during the sampling campaign from 7 to 22 March 2018. [file 13071_2021_4780_MOESM2_ESM.docx]

Table S6: observed *Culicoides* abundance during the sampling campaign from 07 to 22 March 2018. Host diversity is provided. Others hosts refers to swine, poultry and rabbits.

| **Latitude** | **Longitude** | **Date of trapping** | **Cattle** | **Sheep & goats** | **Deer & horses** | **Other hosts** | **Total *Culicoides*** | ***C. bolitinos*** | ***C. enderleini*** | ***C. grahamii*** | ***C. imicola*** | ***C. kibatiensis*** |
| --- | --- | --- | --- | --- | --- | --- | --- | --- | --- | --- | --- | --- |
| -21.045 | 55.7 | 07/03/2018 | 18 | 0 | 0 | 0 | 126 | 9 | 114 | 0 | 3 | 0 |
| -21.093 | 55.736 | 07/03/2018 | 26 | 60 | 0 | 0 | 202 | 15 | 175 | 0 | 12 | 0 |
| -21.097 | 55.755 | 07/03/2018 | 0 | 123 | 0 | 0 | 1106 | 5 | 934 | 9 | 158 | 0 |
| -21.112 | 55.716 | 07/03/2018 | 0 | 90 | 0 | 0 | 8 | 2 | 5 | 0 | 1 | 0 |
| -21.114 | 55.691 | 07/03/2018 | 15 | 0 | 0 | 0 | 584 | 42 | 33 | 5 | 490 | 14 |
| -21.304 | 55.478 | 07/03/2018 | 40 | 0 | 0 | 0 | 46 | 0 | 4 | 0 | 42 | 0 |
| -21.308 | 55.514 | 07/03/2018 | 100 | 0 | 0 | 0 | 16 | 0 | 0 | 0 | 16 | 0 |
| -21.317 | 55.437 | 07/03/2018 | 100 | 0 | 0 | 0 | 8091 | 0 | 214 | 0 | 7877 | 0 |
| -21.346 | 55.53 | 07/03/2018 | 40 | 0 | 0 | 0 | 9 | 0 | 0 | 0 | 0 | 9 |
| -21.358 | 55.538 | 07/03/2018 | 19 | 0 | 0 | 0 | 23 | 2 | 0 | 0 | 13 | 8 |
| -21.014 | 55.496 | 08/03/2018 | 10 | 0 | 0 | 0 | 10 | 0 | 0 | 0 | 3 | 7 |
| -21.017 | 55.544 | 08/03/2018 | 10 | 10 | 0 | 20 | 60 | 0 | 50 | 0 | 10 | 0 |
| -21.025 | 55.507 | 08/03/2018 | 0 | 85 | 0 | 20 | 4 | 0 | 2 | 0 | 1 | 1 |
| -21.03 | 55.471 | 08/03/2018 | 9 | 0 | 0 | 0 | 8 | 0 | 0 | 0 | 0 | 8 |
| -21.039 | 55.462 | 08/03/2018 | 8 | 0 | 0 | 0 | 53 | 0 | 1 | 0 | 0 | 52 |
| -21.046 | 55.526 | 08/03/2018 | 3 | 28 | 0 | 30 | 25 | 0 | 23 | 0 | 0 | 2 |
| -21.061 | 55.527 | 08/03/2018 | 0 | 60 | 0 | 0 | 4 | 0 | 4 | 0 | 0 | 0 |
| -21.214 | 55.53 | 08/03/2018 | 50 | 0 | 0 | 0 | 19 | 1 | 0 | 0 | 8 | 10 |
| -21.257 | 55.51 | 08/03/2018 | 38 | 0 | 0 | 0 | 7 | 0 | 0 | 0 | 7 | 0 |
| -21.266 | 55.536 | 08/03/2018 | 49 | 0 | 0 | 0 | 7 | 0 | 0 | 2 | 5 | 0 |
| -21.267 | 55.484 | 08/03/2018 | 14 | 0 | 0 | 0 | 17 | 0 | 0 | 0 | 4 | 13 |
| -21.269 | 55.491 | 08/03/2018 | 60 | 0 | 0 | 0 | 155 | 0 | 3 | 0 | 152 | 0 |
| -20.951 | 55.686 | 09/03/2018 | 1 | 50 | 0 | 20 | 8 | 0 | 7 | 0 | 1 | 0 |
| -21.037 | 55.668 | 09/03/2018 | 0 | 75 | 0 | 0 | 4 | 0 | 0 | 0 | 4 | 0 |
| -21.072 | 55.72 | 09/03/2018 | 0 | 55 | 0 | 0 | 22 | 5 | 9 | 0 | 8 | 0 |
| -21.231 | 55.469 | 09/03/2018 | 35 | 60 | 0 | 0 | 52 | 0 | 0 | 0 | 52 | 0 |
| -21.242 | 55.476 | 09/03/2018 | 25 | 0 | 0 | 0 | 56 | 13 | 0 | 0 | 35 | 8 |
| -21.099 | 55.661 | 12/03/2018 | 60 | 0 | 0 | 0 | 2507 | 73 | 0 | 27 | 534 | 1873 |
| -21.129 | 55.647 | 12/03/2018 | 0 | 150 | 0 | 20 | 12 | 0 | 0 | 0 | 10 | 2 |
| -21.133 | 55.606 | 12/03/2018 | 0 | 70 | 0 | 50 | 17 | 0 | 0 | 3 | 0 | 14 |
| -21.142 | 55.587 | 12/03/2018 | 46 | 0 | 0 | 0 | 28 | 0 | 0 | 0 | 0 | 28 |
| -21.158 | 55.633 | 12/03/2018 | 11 | 0 | 0 | 0 | 395 | 0 | 0 | 0 | 0 | 395 |
| -21.221 | 55.444 | 12/03/2018 | 1 | 0 | 0 | 0 | 9 | 0 | 0 | 0 | 9 | 0 |
| -21.243 | 55.439 | 12/03/2018 | 23 | 0 | 0 | 0 | 18 | 0 | 0 | 0 | 18 | 0 |
| -21.257 | 55.427 | 12/03/2018 | 20 | 0 | 0 | 0 | 215 | 0 | 82 | 0 | 133 | 0 |
| -21.358 | 55.54 | 12/03/2018 | 20 | 0 | 0 | 0 | 44 | 0 | 0 | 0 | 40 | 4 |
| -21.205 | 55.554 | 13/03/2018 | 10 | 0 | 0 | 0 | 10 | 0 | 0 | 0 | 0 | 10 |
| -21.209 | 55.529 | 13/03/2018 | 60 | 0 | 0 | 0 | 13 | 5 | 0 | 3 | 0 | 5 |
| -21.216 | 55.545 | 13/03/2018 | 4 | 0 | 0 | 0 | 10 | 3 | 0 | 1 | 0 | 6 |
| -21.219 | 55.334 | 13/03/2018 | 96 | 0 | 0 | 75 | 51 | 0 | 0 | 0 | 0 | 51 |
| -21.234 | 55.35 | 13/03/2018 | 31 | 0 | 0 | 0 | 145 | 9 | 5 | 0 | 0 | 131 |
| -21.234 | 55.525 | 13/03/2018 | 26 | 0 | 0 | 0 | 0 | 0 | 0 | 0 | 0 | 0 |
| -21.236 | 55.349 | 13/03/2018 | 1 | 80 | 0 | 28 | 386 | 0 | 0 | 3 | 203 | 180 |
| -21.236 | 55.559 | 13/03/2018 | 50 | 0 | 0 | 0 | 23 | 2 | 0 | 0 | 7 | 14 |
| -21.239 | 55.353 | 13/03/2018 | 0 | 100 | 0 | 0 | 33 | 0 | 0 | 0 | 30 | 3 |
| -21.244 | 55.532 | 13/03/2018 | 16 | 0 | 0 | 0 | 490 | 112 | 0 | 7 | 133 | 238 |
| -21.249 | 55.512 | 13/03/2018 | 14 | 0 | 0 | 0 | 93 | 6 | 0 | 0 | 63 | 24 |
| -21.279 | 55.379 | 13/03/2018 | 0 | 99 | 0 | 0 | 7137 | 0 | 1584 | 0 | 5553 | 0 |
| -21.132 | 55.331 | 14/03/2018 | 50 | 0 | 0 | 0 | 12 | 0 | 0 | 1 | 0 | 11 |
| -21.133 | 55.338 | 14/03/2018 | 7 | 0 | 0 | 0 | 22 | 0 | 0 | 0 | 8 | 14 |
| -21.167 | 55.355 | 14/03/2018 | 21 | 0 | 0 | 0 | 678 | 0 | 0 | 0 | 0 | 678 |
| -21.237 | 55.318 | 14/03/2018 | 0 | 150 | 1 | 0 | 126 | 4 | 0 | 0 | 100 | 22 |
| -21.29 | 55.548 | 14/03/2018 | 88 | 0 | 0 | 0 | 84 | 0 | 0 | 0 | 36 | 48 |
| -21.304 | 55.558 | 14/03/2018 | 35 | 0 | 0 | 0 | 1403 | 1050 | 0 | 0 | 46 | 307 |
| -21.318 | 55.562 | 14/03/2018 | 18 | 0 | 0 | 0 | 780 | 720 | 0 | 0 | 21 | 39 |
| -21.331 | 55.573 | 14/03/2018 | 71 | 0 | 0 | 0 | 5 | 0 | 0 | 0 | 5 | 0 |
| -21.062 | 55.328 | 15/03/2018 | 13 | 0 | 0 | 0 | 1645 | 115 | 0 | 10 | 0 | 1520 |
| -21.091 | 55.337 | 15/03/2018 | 13 | 0 | 0 | 0 | 206 | 0 | 0 | 0 | 0 | 206 |
| -21.097 | 55.329 | 15/03/2018 | 15 | 0 | 0 | 0 | 4 | 0 | 0 | 0 | 4 | 0 |
| -21.105 | 55.325 | 15/03/2018 | 30 | 40 | 12 | 41 | 852 | 8 | 0 | 20 | 16 | 808 |
| -21.105 | 55.325 | 15/03/2018 | 30 | 40 | 12 | 41 | 406 | 9 | 0 | 5 | 0 | 392 |
| -21.126 | 55.328 | 15/03/2018 | 10 | 0 | 0 | 0 | 609 | 0 | 0 | 7 | 15 | 587 |
| -21.17 | 55.559 | 15/03/2018 | 80 | 0 | 0 | 0 | 1 | 0 | 0 | 0 | 0 | 1 |
| -21.189 | 55.554 | 15/03/2018 | 60 | 0 | 0 | 0 | 0 | 0 | 0 | 0 | 0 | 0 |
| -21.205 | 55.567 | 15/03/2018 | 20 | 0 | 0 | 0 | 0 | 0 | 0 | 0 | 0 | 0 |
| -21.207 | 55.596 | 15/03/2018 | 50 | 0 | 0 | 0 | 0 | 0 | 0 | 0 | 0 | 0 |
| -21.301 | 55.539 | 15/03/2018 | 3 | 0 | 0 | 0 | 144 | 78 | 0 | 0 | 36 | 30 |
| -20.967 | 55.36 | 16/03/2018 | 0 | 12 | 0 | 15 | 155 | 0 | 0 | 0 | 145 | 10 |
| -21.029 | 55.331 | 16/03/2018 | 9 | 0 | 0 | 0 | 183 | 30 | 7 | 0 | 33 | 113 |
| -21.048 | 55.334 | 16/03/2018 | 0 | 30 | 30 | 0 | 5173 | 198 | 0 | 28 | 68 | 4879 |
| -21.057 | 55.267 | 16/03/2018 | 40 | 0 | 0 | 0 | 2295 | 37 | 219 | 0 | 2002 | 37 |
| -21.097 | 55.262 | 16/03/2018 | 20 | 0 | 0 | 0 | 173 | 61 | 4 | 0 | 108 | 0 |
| -21.205 | 55.594 | 16/03/2018 | 57 | 0 | 0 | 0 | 1 | 0 | 0 | 0 | 0 | 1 |
| -21.219 | 55.597 | 16/03/2018 | 1 | 120 | 0 | 0 | 742 | 0 | 0 | 0 | 0 | 742 |
| -21.235 | 55.581 | 16/03/2018 | 95 | 0 | 0 | 0 | 35 | 0 | 0 | 0 | 9 | 26 |
| -21.235 | 55.598 | 16/03/2018 | 3 | 0 | 0 | 0 | 13 | 0 | 0 | 0 | 0 | 13 |
| -21.26 | 55.599 | 16/03/2018 | 65 | 0 | 0 | 0 | 103 | 0 | 0 | 0 | 0 | 103 |
| -21.305 | 55.63 | 19/03/2018 | 55 | 0 | 0 | 0 | 120 | 0 | 0 | 0 | 0 | 120 |
| -21.332 | 55.652 | 19/03/2018 | 26 | 0 | 0 | 0 | 43 | 28 | 0 | 7 | 1 | 7 |
| -21.346 | 55.668 | 19/03/2018 | 50 | 0 | 0 | 0 | 108 | 104 | 0 | 0 | 2 | 2 |
| -21.367 | 55.731 | 19/03/2018 | 50 | 0 | 0 | 0 | 1106 | 653 | 360 | 13 | 80 | 0 |
| -21.369 | 55.639 | 19/03/2018 | 25 | 0 | 0 | 0 | 14 | 7 | 0 | 0 | 7 | 0 |
| -20.908 | 55.505 | 20/03/2018 | 0 | 24 | 0 | 0 | 81 | 0 | 12 | 0 | 69 | 0 |
| -20.926 | 55.5 | 20/03/2018 | 13 | 0 | 0 | 0 | 49 | 0 | 7 | 0 | 42 | 0 |
| -20.927 | 55.656 | 20/03/2018 | 3 | 30 | 0 | 0 | 5 | 0 | 4 | 0 | 1 | 0 |
| -20.928 | 55.459 | 20/03/2018 | 14 | 0 | 0 | 0 | 5796 | 0 | 4 | 0 | 5792 | 0 |
| -20.963 | 55.673 | 20/03/2018 | 0 | 200 | 0 | 50 | 0 | 0 | 0 | 0 | 0 | 0 |
| -21.283 | 55.388 | 20/03/2018 | 70 | 0 | 0 | 0 | 4373 | 480 | 1440 | 0 | 2453 | 0 |
| -21.327 | 55.614 | 20/03/2018 | 45 | 0 | 0 | 0 | 54 | 15 | 0 | 0 | 8 | 31 |
| -21.336 | 55.615 | 20/03/2018 | 40 | 0 | 0 | 0 | 43 | 17 | 0 | 0 | 0 | 26 |
| -21.34 | 55.591 | 20/03/2018 | 20 | 0 | 0 | 0 | 23 | 6 | 0 | 0 | 6 | 11 |
| -21.358 | 55.61 | 20/03/2018 | 55 | 0 | 0 | 0 | 238 | 147 | 0 | 0 | 14 | 77 |
| -21.381 | 55.608 | 20/03/2018 | 30 | 0 | 0 | 0 | 66 | 7 | 0 | 0 | 59 | 0 |
| -20.9 | 55.558 | 21/03/2018 | 0 | 34 | 0 | 0 | 4 | 4 | 0 | 0 | 0 | 0 |
| -20.953 | 55.615 | 21/03/2018 | 9 | 45 | 0 | 0 | 14 | 0 | 6 | 0 | 8 | 0 |
| -20.954 | 55.571 | 21/03/2018 | 0 | 0 | 400 | 0 | 36 | 0 | 0 | 1 | 35 | 0 |
| -20.988 | 55.636 | 21/03/2018 | 3 | 16 | 0 | 0 | 17 | 3 | 2 | 1 | 9 | 2 |
| -20.99 | 55.647 | 21/03/2018 | 0 | 110 | 0 | 0 | 0 | 0 | 0 | 0 | 0 | 0 |
| -21.154 | 55.437 | 21/03/2018 | 3 | 0 | 0 | 0 | 17 | 1 | 0 | 0 | 8 | 8 |
| -21.14 | 55.487 | 22/03/2018 | 1 | 0 | 0 | 3 | 65 | 1 | 0 | 1 | 4 | 59 |
| -21.148 | 55.44 | 22/03/2018 | 10 | 0 | 0 | 3 | 46 | 0 | 0 | 0 | 23 | 23 |
|  |  |  |  |  |  | **Total** | **50526** | **4087** | **5314** | **154** | **26908** | **14063** |
